# Hsp90α promotes lipogenesis by stabilizing FASN and promoting FASN transcription via LXRα in hepatocellular carcinoma

**DOI:** 10.1016/j.jlr.2024.100721

**Published:** 2024-12-05

**Authors:** Zihao Deng, Lixia Liu, Guantai Xie, Zhenming Zheng, Jieyou Li, Wenchong Tan, Yaotang Deng, Jinxin Zhang, Manfeng Liang, Yingxia Wu, Zhifeng Zhou, Yan Li, Yukui Chen, Yaling Huang, Hairou Su, Guibing Wu, Xiongjie Shi, Shengpei Cen, Yandan Liao, Yilin Liu, Fei Zou, Xuemei Chen

**Affiliations:** 1 Department of Occupational Health and Medicine, Guangdong Provincial Key Laboratory of Tropical Disease Research, School of Public Health, Southern Medical University, Guangzhou, China; 2 Department of Hygiene Inspection and Quarantine Science, School of Public Health, Southern Medical University, Guangdong Provincial Key Laboratory of Tropical Disease Research, Guangzhou, China

**Keywords:** HCC, lipid accumulation, Hsp90α, Hsp90 inhibitor, FASN, SREBP1, LXRα

## Abstract

Excessive lipid accumulation promotes the occurrence and progression of hepatocellular carcinoma (HCC), accompanied by high levels of fatty acid synthetase (FASN) and more active lipogenesis. Heat shock protein 90 (Hsp90) acts as a chaperone to maintain the stability and activity of the client proteins. Studies have revealed that Hsp90 regulates the lipid metabolism of HCC, but the effect of Hsp90 on FASN still remains unknown. This study aims to discover the mechanism of Hsp90 inhibition on lipid accumulation and investigate the different effects of Hsp90 N-terminal domain inhibitor STA9090 and C-terminal domain inhibitor novobiocin on FASN protein stability and transcription pathway in HCC. We found that HCC cells tended to store lipids, which could be disrupted by Hsp90 inhibitors in vivo and in vitro. High levels of Hsp90α and FASN in tumor tissue had correlation with poor prognosis of HCC patients, and Hsp90α interacted with FASN to maintain its protein stability. Furthermore, N-terminal domain of Hsp90α was essential for process of sterol regulatory element binding protein 1 to activate FASN transcription and Hsp90α prevented proteasomal degradation of liver X receptor α to upregulate FASN transcription via liver X receptor α/sterol regulatory element binding protein 1 axis. Our data reveal that Hsp90α promotes lipid accumulation by increasing the protein stability and FASN mRNA transcription, and can be alleviated by Hsp90 inhibitors, which provides a theoretical basis for Hsp90-targeted therapy on lipid metabolism in HCC.

Based on the 2022 global cancer data, there were 865,269 new cases of liver cancer, ranking sixth among all cancers, while the deaths reached 757,948 ranking third among all cancers (1). Hepatocellular carcinoma (HCC) is the most common type of primary liver cancer, accounting for approximately 90% of all cases. The current treatment options for HCC include surgical resection, liver transplantation, radiofrequency ablation (RFA), transarterial chemoembolization (TACE), and targeted therapy. The main challenges in HCC treatment are high recurrence rate after surgery or transplantation and drug resistance. Though combination therapies and personalized medicine approaches have been used to improve existing treatment strategies, more research is needed to understand the molecular mechanisms underlying HCC development and progression, which could lead to the discovery of new targets for therapy (2, 3, 4).

HCC is mainly developed from chronic liver disease caused by viral hepatitis (5). However, the incidence of HCC caused by lipid metabolism disorder including obesity, non-alcoholic liver disease and diabetes is rapidly increasing (6, 7). It is observed that lipid synthesis, lipid uptake and storage are often upregulated in tumor cells (8, 9). The activation of cancer-promoting signaling pathways is usually coexisted with changes in the expression and activity of enzymes related to lipid metabolism (10). Fatty acid synthetase (FASN) is a key enzyme in cellular de novo fatty acid (FA) synthesis, highly expressing in HCC samples (11), and is transcriptionally activated by its upstream transcription factor liver X receptor (LXR) as well as sterol regulatory element binding protein 1 (SREBP1) (12). FASN takes charge of catalyzing the generation of 16-carbon palmitate from malonyl-CoA and acetyl-CoA. Additionally, palmitate is one of the main components of cell membranes and can be incorporated into triglyceride (TG) for energy storage as lipid accumulation.

As a molecular chaperone, heat shock protein 90 (Hsp90) has the highly conserved expression and is found overexpressed in tumor cells, which account for more than 4% of the total cellular protein (13, 14). Hsp90 monomer includes three domains according to different spatial positions and molecular functions. The ATP binding site on N-terminal domain (NTD) is the structural basis of the ATPase activity of Hsp90, which enables Hsp90 to briefly dimerize through this domain and bind to its substrate protein, thereby exerting the function of molecular chaperone (15, 16). Middle domain (MD) can promote ATP hydrolysis and participate in co-chaperones of molecules and the binding of some substrate proteins (17). C-terminal domain (CTD) is the basis for mediating Hsp90 monomer to form its homologous dimer form with molecular chaperone function and combines with hundreds of client proteins (18).

Since Hsp90 participates in the activation and stability maintenance of a variety of proteins by assisting the folding or degradation of hundreds of client proteins, Hsp90 has a significant impact in signal transduction, proliferation, and survival of cancer cells (19), emerging as a potential target available for cancer therapy (20). Due to the different domains of Hsp90 have their respective functions, the currently developed Hsp90 inhibitors can be classified as targeting either the NTD or the CTD. STA9090, as a N-terminal inhibitor, was one of the very few inhibitors of Hsp90 that become a candidate of phase III clinical trials, had been shown to selectively target the more active Hsp90 in tumor cells, but having a lesser effect on nontumor cells (21, 22). However, many of these N-terminal inhibitors have been stopped at different phases of clinical trials and failed to be approved by the Food and Drug Administration due to the negative effects of heat shock response (23). Other Hsp90 inhibitors targeting CTD without inducing heat shock response also are under investigation. For example, Novobiocin (NB) is a C-terminal inhibitor of Hsp90, which causes abnormal conformation changes of Hsp90 dimer, indirectly interfering the N-terminal ATPase activity (24). Recently, TAS-116 was shown to have selective effect on inhibiting cytosolic Hsp90, downregulating substrate proteins of Hsp90 in xenograft models (25) and had been marketed for the therapy of gastrointestinal stromal tumors as an oral Hsp90 inhibitor in Japan. This gives us new hope for the possibility to bring Hsp90 inhibitors to clinical applications.

More and more findings confirm the link between Hsp90 and cellular lipid metabolism. It was reported that Hsp90 showed increased expression in alcoholic liver of human and mouse, and the Hsp90 inhibitor 17-DMAG was found to reduce liver injury in mouse alcoholic liver model (26). Furthermore, previous studies have proved that Hsp90 promoted de novo FA synthesis by maintaining the protein stability of SREBP1 (27), Hsp90 inhibitor geldanamycin analogs exerted antiadipogenic effects by simultaneously inhibiting mineralocorticoid, glucocorticoid receptors and peroxisome proliferator-activated receptor γ activities (28, 29). Since the complexity of lipid metabolism and Hsp90 itself, more studies need to be done to reveal the detailed mechanisms.

In this study, we find that Hsp90α promotes lipid accumulation by increasing FASN protein stability and mRNA transcription, and can be alleviated by Hsp90 inhibitors via LXRα-SREBP1 axis, which reveals a mechanism for potential Hsp90-targeted therapy on lipid metabolism in HCC.

## Materials and Methods

### Ethics statement

All human tissue samples were collected under the guidelines of the Declaration of Helsinki. All patients offered written informed consents and the protocol (Approval Code: NFEC-2022-184) was approved by the Nanfang Hospital Ethics Committee (Guangzhou, China).

### Human tissue

HCC tissue and adjacent normal tissue were collected and analyzed from 10 HCC patients during surgery at Nanfang Hospital (Guangzhou, China). Prior to surgery, all patients had not received chemotherapy or radiotherapy.

### Cell transfection

All siRNA were purchased from GenePharma (Shanghai, China). Plasmids of CRISPR-Cas9 contained sgRNA for Hsp90α KO were purchased from iGene Biotechnology (Guangzhou, China). For further details about CRISPR-Cas9 plasmids and sgRNA design, please refer to the Supplemental Material and Methods. All siRNA and sgRNA sequences were showed in Supplemental Table S1. The full-length of Hsp90α (hemagglutinin (HA)-tagged full-length), Hsp90α NTD deletion (HA-tagged MD-CTD, ΔN), CTD deletion (HA-tagged NTD-MD, ΔC), and FLAG-Hsp90α plasmids were kindly provided by Matthias Mayer (ZMBH, Heidelberg University, Germany). The siRNA, sgRNA, or plasmids were transfected with Lipofectamine 3000 (Invitrogen, CA) as described previously (30) and confirmed by Western blotting.

### Cell culture and reagents

LO2 (GNHu6), HepG2 (SCSP-510), and Huh7 (SCSP-526) cell lines were obtained from the Cell Bank, Chinese Academy of Medical Sciences (Shanghai, China). KO-Hsp90α HepG2 cell line was constructed by sgRNA as described previously (30). Each cell line was authenticated and the test results of Mycoplasma Detection Kit (Yeasen, Shanghai, China) showed no mycoplasma contamination. Cells were maintained in a 5% CO2 humidiﬁed atmosphere at 37°C, using DMEM (Gibco, MA), supplemented by 10% FBS (Gibco) and 100 U/ml penicillin-streptomycin mixed solution (CA0075, Leagene, Beijing, China). STA9090 (S1159), NB (S2492) and MG132 (S2619) were acquired from SelleckChem (Houston, TX). Cycloheximide (CHX) was acquired from Sigma-Aldrich (C7698, MO).

### Xenotransplantation experiments

In vivo experiments were proceeded under the Animal Welfare of Southern Medical University (Guangzhou, China) and obtained permission of the Animal Care and Utilization Committee of Southern Medical University (Approval code L2020029). Male BALB/c nude mice (4 weeks of age, 15–20 g) were gained from the Laboratory Animal Center of Southern Medical University. The nude mice were fed with standard laboratory diet in specific pathogen-free room at 25°C ± 1°C with a 12 h light/dark schedule. The dorsal side of the nude mice was injected subcutaneously with HepG2 cells (1 × 10^7^) resuspended in DMEM (100 μl, serum-free). On the 10th day after the cell injection, the nude mice were randomly assigned to 3 groups (n = 4 mice per group) according to different treatments: (i) STA9090 group (5 μl/g intraperitoneally injected with STA9090 (25 mg/kg, 10% DMSO, 18% Cremophor RH (MB2572, Sigma-Aldrich), 3.6% glucose (158968, Sigma-Aldrich), 68.4% ddH2O)); (ii) NB group (5 μl/g intraperitoneally injected with NB (50 mg/kg, 10% DMSO, 18% Cremophor RH, 3.6% glucose, and 68.4% ddH2O)); (iii) control group was treated in the same way but without STA9090 or NB. The injection was performed every other day for 10 days. The body weight of the nude mice as well as the volume of each xenograft tumor were detected every two days. After that, all the nude mice were sacrificed by cervical dislocation followed by extraction of the xenograft tumors. The volume of xenograft tumors was acquired using the following formula: longest diameter × (shortest diameter) ^2^ × 0.5.

### Immunohistochemistry

Immunohistochemistry (IHC) was carried out under the guidance of the standard labeled streptavidin-biotin complex protocol (BOSTER, Guangzhou, China) with antibodies against Hsp90α (1:10,000) (6251422, Enzo, NY), FASN (1:200) (10624-2-AP, Proteintech, Wuhan, China). Immunoglobulin G of corresponding species was used as negative controls. Statistical analysis of normalized expression of FASN in the xenograft tumor of nude mice and HCC tissues and adjacent nontumoral tissues of HCC patients were detected by Image-Pro Plus 6.0 (https://www.scientific-computing.com/search/node?keys=Image-Pro+Plus) (Media Cybernetics, MD).

### Nile red fluorescent staining

Nile red (NR) fluorescent dye (N8440, Solarbio, Beijing, China) that selectively stains neutral lipid was applied to detect the intracellular lipid accumulation (31, 32). For fluorescent microscope, cells were seeded using confocal dish and processed treatment, then fixed with 1.5% glutaraldehyde (G5882, Sigma-Aldrich) (room temperature, 20 min), and permeabilized using 0.1% Triton X-100 (MB2486, MeilunBio, Dalian, China) (room temperature, 15 min). After that, cells were stained with 1 μg/ml NR (dissolved in PBS, 37°C, 15 min). For xenograft tumors, tumors were frozen and sliced, then stained with 1 μg/ml NR (dissolved in PBS, 37°C, 15 min). Fluorescent images were acquired with Olympus FV1000 Confocal Laser Scanning Microscope (Tokyo, Japan) and measured using ImageJ v1.8.0 (NIH, MD). For flow cytometry, cells were digested and deposited in 1.5 ml tubes by centrifugation. Cells were then resuspended and stained with 1 μg/ml NR (dissolved in PBS, 37°C, 15 min). Guava Flow Cytometry System (6HT2L, Millipore, MA) was applied to detect the cellular NR fluorescent signal. FlowJo V10 (https://www.flowjo.com/solutions/flowjo) (BD, OR, USA) was used to visualize the flow cytometry data.

### Determination of triglyceride

The TG assay kits (A110-1-1, Nanjing Jiancheng Bioengineering Institute, Nanjing, China) was applied to measure the intracellular TG content. Cells were seeded into 35 mm cell culture dish. After processing, the cells were lysed in 1% Triton X-100 solution (dissolved in PBS, 4°C, 30 min). Ultrasonic system JY88-Ⅱ (Scientz, Ningbo, China) was applied to obtain more fragmentized cells. The levels of TG were examined by the Spark Multimode Microplate reader (Tecan, Männedorf, Switzerland) and normalized by total protein levels.

### RNA-seq

Trizol (15596018, Thermo Fisher Scientific, MA) was applied to obtain RNA from HepG2 cells. NEBNext UltraTM RNA Library Prep Kit (E7490, New England Biolabs, MA) was used for library construction, and the library was purified with AMPure XP system beads (A-A63880, Beckman Coulter, IN). RNA sequencing was performed using NovaSeq 6000 (Illumina, CA). Spliced Transcripts Alignment to a Reference (STAR, CSHL) was used for alignment between clean reads and reference genome.

### Gene Ontology pathway enrichment

Significantly altered genes (*P* < 0.05) and proteins (FC < 0.85 or FC > 1.15, *P* < 0.05) in the RNA-seq and proteomics data of HepG2 cells from different treatment group were uploaded to the DAVID (https://david.ncifcrf.gov/) and classified as biological process and molecular function.

### Western blotting and co-immunoprecipitation

Western blot analysis was carried out as described previously (30). The antibodies were listed in Supplemental Table S2. Protein blots were scanned using Li-COR Odyssey Imaging scanner (Li-COR Biosciences, NE) and quantified using ImageJ v1.8.0 (https://imagej.en.softonic.com/?ex=RAMP-2639.2) (NIH, MD). For co-immunoprecipitation (Co-IP), 1 μg antibodies were incubated with 1000 μg protein extracts from HCC cells (4°C, overnight). After that, Protein A + G Magnetic Beads (P2108, Beyotime, Shanghai, China) were mixed with the protein extracts (20 μl/ml, 4°C, 3 h) and purified by magnetic frame. The immune complexes were boiled, and then isolated by SDS-polyacrylamide gels, and the bands were tested by the indicated antibodies.

### Protein half-life assay

HCC cell lines WT/Hsp90α-KO (HepG2, Huh7) were treated with 25 μg/ml CHX (C7698, Sigma-Aldrich, MO) for indicated times. Hsp90 inhibitors were added to the medium 1 h after CHX treatment. Protein half-life was then detected by Western blotting.

### RNA isolation and real-time PCR

Total RNA of cells was acquired using AG RNAex Pro reagent (AG21102, Accurate Biology, Hunan, China). Reverse transcription of RNA was carried out by PrimeScript RT Reagent kit supplemented with gDNA Eraser (CRR047A, Takara, Tokyo, Japan), followed by RT-PCR using Hieff qPCR SYBR Green Master Mix (No Rox) (Yeasen). The primers for PCR were exhibited in Supplemental Table S3. Reaction conditions were set under the manufacturer’s recommendations.

### Chromatin immunoprecipitation

Crosslinking between DNA and protein was performed using 1.42% formaldehyde in DMEM (room temperature, 15 min) and terminated using 0.125 M glycine (room temperature, 5 min). Chromatin was diluted in chromatin immunoprecipitation (ChIP) buffer according to the published protocol for the fast chromatin immunoprecipitation method (33). Ultrasonic system JY88-Ⅱ (Scientz, Ningbo, China) was applied to obtain 100-600 bp DNA segments verified by agarose gel electrophoresis. A total of 100 μl of chromatin was transferred as input. Briefly, 1 μg antibodies were mixed with the chromatin (4°C, overnight). After that, Protein A + G Magnetic Beads (P2108, Beyotime, Shanghai, China) were mixed with the chromatin (20 μl/ml, 4°C, 3 h) and purified by magnetic frame. Subsequently, 10 % Chelex 100 (1421253, Bio-Rad, CA) was swirled with magnetic beads (100°C, 10 min). After decrosslinking by protease K (55°C, 0.5 h), the samples were heated (100°C, 10 min) to inactivate the protease K. The DNA samples were amplified by PCR and detected by agarose gel electrophoresis. The ChIP PCR primers were listed in Supplemental Table S4.

### Dual-luciferase reporter gene assay

WT/Hsp90α-KO HepG2 cells in 35 mm cell culture dish were transfected with LXRE luciferase reporter vector plasmid (11574ES03, Yeasen Biotechnology) and renilla luciferase plasmid (pGL3-Renilla) with Lipofectamine 3000 (Invitrogen) for 30 h. After that, Reporter Gene Assay Kit (RG027, Beyotime Biotechnology, Shanghai, China) was adopted to conduct the dual-luciferase assays. The expression of firefly luciferase and renilla luciferase was reflected by the Spark Multimode Microplate reader (Tecan, Männedorf, Switzerland). The luminescence of firefly luciferase was normalized by luminescence of renilla luciferase.

### Immunofluorescence assays

Cells were seeded onto confocal dishes for indicated treatment, then fixed in 4% paraformaldehyde (room temperature, 15 min), followed by permeating in prechilled methanol (−20°C, 10 min), and blocked using 3% BSA (dissolve in PBS, room temperature, 0.5 h). The antibodies were listed in Supplemental Table S5 and used to incubate with the cells (4°C, overnight) and then combined with corresponding second antibodies linked with fluorescent dyes (room temperature, 2 h). 4',6-diamidino-2-phenylindole was used for coloration of cell nucleus (room temperature, 5 min). Fluorescent images acquired with Olympus FV1000 Confocal Laser Scanning Microscope (Tokyo, Japan) were measured using ImageJ v1.8.0 (NIH).

### Cytoplasmic-nuclear isolation

The separation and purification of cytoplasmic and nuclear proteins was carried out by the cytoplasmic-nuclear isolation kit (KGP1100, HeyGen BioTECH, China). Briefly, 450 μl Buffer A and 50 μl Buffer B (17 μl 100 mM PMSF and 0.45 μl protease inhibitor) were mixed in 100 mm dish (4°C, 30 min). After centrifuge the cell lysate (3000 rpm, 4°C, 10 min), supernatants (cytoplasmic protein) were collected in precooled tubes. Subsequently, 100 μl Buffer C (17 μl 100 mM PMSF, 0.1 μl protease inhibitor) was used to lyse the pellet (nucleus), and the tubes were shaken with a vortex mixer (maximum speed, 15 s), then placed on ice for 30–60 min. Shaking vigorously for 15 s every 10 min. Supernatants (nucleoprotein) were centrifuged (14,000 g, 4°C, 30 min) and transfer to precooled tubes as soon as possible. BCA method was performed for the protein quantification of the cytoplasmic and nuclear proteins extracted above.

### Transmission electron microscopy

Sample preparation was carried out as described previously (34). HCC xenograft tumors tissue (less than 1 mm^3^) from Ctrl and STA9090 group mice were fixed in 2.5% glutaraldehyde (pH = 7.4, room temperature, 1 h) and then 2% osmium tetroxide (room temperature, 1 h). Gradual dehydration was carried out by ethanol (30%, 50%, 70%, 80%, 90%, and 100%) for 10 min each time and then soaked in propylene oxide for 10 min, followed by encapsulating with pure epoxy resin (72°C, 8 h). Ultrathin sections were stained with uranyl acetate and lead citrate and loaded into a 200-mesh copper mesh. Ultrastructural images of the Golgi were obtained using transmission electron microscope (Hitachi-7500, Hitachi, Tokyo, Japan) at 60 kV.

### Statistical analysis

HCC patients were divided into high expression group and low expression group according to the median values of different genes, and method of Kaplan-Meier was used to calculate the overall survival rate. The results were shown as mean ± SD. All data were acquired from three independent experiments, with at least three replicates or counts per group in each experiment. Statistical analyses were carried out by SPSS 21.0 software (https://www.ibm.com/products/spss-statistics) (IBM, IL, USA). Two-tailed Student’s *t* test was performed to analyze the *P* values. *p* < 0.05 was considered as statistically significant.

## Results

### HCC cell lines presented with higher lipid accumulation than hepatocyte cell line

**Fig. 1:**
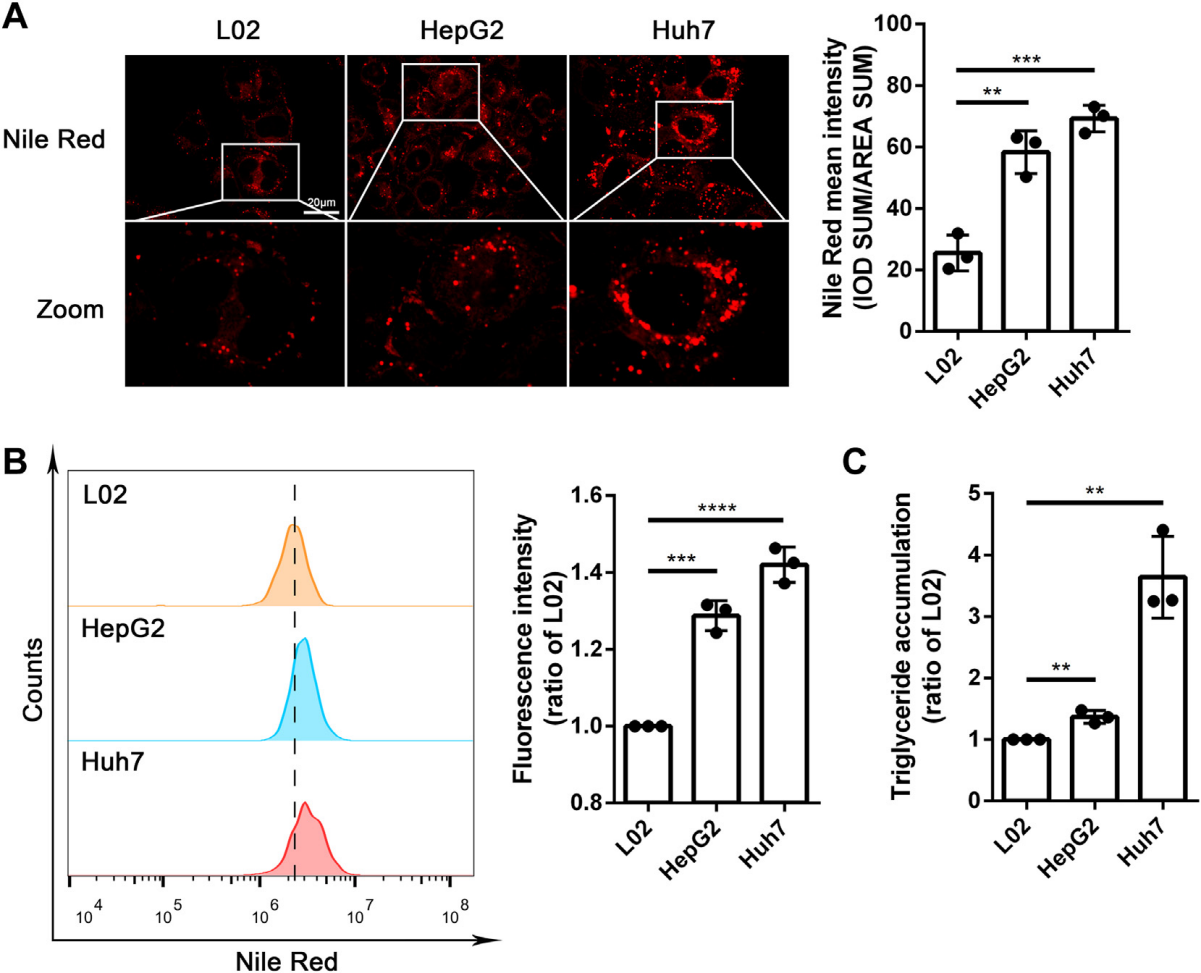
Liver cancer cell lines have more lipid accumulation than normal liver cell line. A: Representative cells fluorescence images (*left*) and quantification (*right*) of Nile red staining in HCC cell lines HepG2, Huh7, and hepatocyte cell line L02 were shown. Scale bar: 20 μm. Data are shown as means ± SD, n = 3 per group, ∗∗*P* < 0.01, ∗∗∗*P* < 0.001. B: The lipid accumulation levels were detected by flow cytometry with Nile *red*. Data are shown as means ± SD, n = 3 per group, ∗∗∗*P* < 0.001, ∗∗∗∗*P* < 0.0001. C: The cellular TG levels were measured by assay kits. Data are shown as means ± SD, n = 3 per group, ∗∗*P* < 0.01. HCC, hepatocellular carcinoma; TG, triglyceride.

Most cells store FAs in cytosolic lipid droplets (LDs) (35). TG is the main component of intracellular LD, consisting of a glycerol backbone and three FAs attached to it and can be stained with NR dye as neutral lipid (36). To detect the lipid content of the cells, we used NR staining for LD detection to show the difference of lipid accumulation in liver cell line L02, HCC cell lines HepG2 and Huh7. After NR staining, both the confocal laser microscope images (Fig. 1A) and the results of flow cytometry (Fig. 1B) showed that HCC cells had more lipid accumulation than normal liver cell line L02. Besides, the elevated intracellular TG content in HCC cell lines was also consistent with the increased lipid accumulation detected by NR (Fig. 1C).

### Hsp90 inhibitors reduce the lipid accumulation in cell lines and xenotransplantation model of HCC in nude mouse

**Fig. 2:**
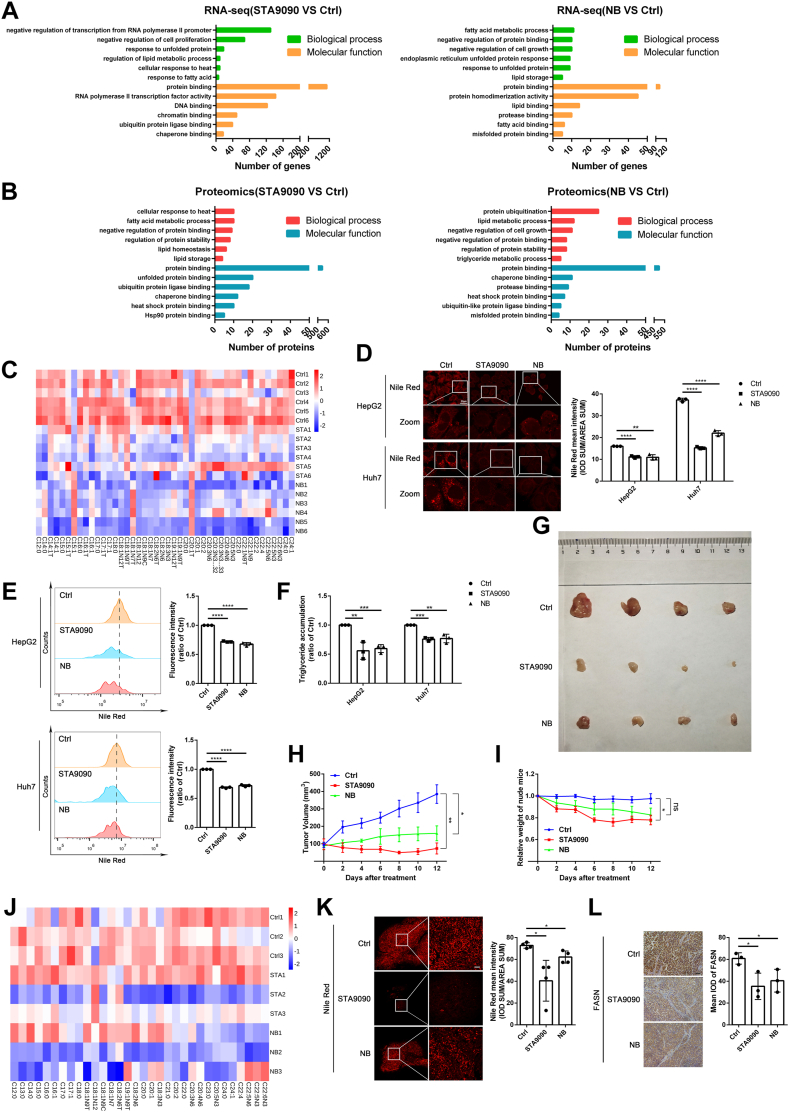
Hsp90 inhibitor reduces the lipid accumulation in HCC in vivo and in vitro. A: Biological process and cellular function enrichment analysis of genes extracted from HepG2 cells treated with DMSO (1 μl DMSO in 1 ml DMEM for 24 h) or STA9090 (100 nM for 24 h) or NB (500 μM for 24 h). B: Biological process and cellular function enrichment analysis of proteins extracted from HepG2 cells treated with DMSO (1 μl DMSO in 1 ml DMEM for 24 h) or STA9090 (100 nM for 24 h) or NB (500 μM for 24 h). C: Heatmap analysis of 44 fatty acid species in HepG2 cells treated with DMSO (1 μl DMSO in 1 ml DMEM for 24 h) or STA9090 (100 nM for 24 h) or NB (500 μM for 24 h) was shown. D: Fluorescence images and quantification of Nile *red* staining in HepG2 and Huh7 were shown. Scale bar: 20 μm. Data are shown as means ± SD, n = 3 per group, ∗∗*P* < 0.01, ∗∗∗∗*P* < 0.0001. E: The lipid accumulation levels of HepG2 and Huh7 cells treated with DMSO (1 μl DMSO in 1 ml DMEM for 24 h) or STA9090 (100 nM for 24 h) or NB (500 μM for 24 h) were detected by flow cytometry with Nile *Red*. Data are shown as means ± SD, n = 3 per group, ∗∗∗∗*P* < 0.0001. F: The cellular TG levels of HepG2 and Huh7 cells were measured. Data are shown as means ± SD, n = 3 per group, ∗∗*P* < 0.01, ∗∗∗*P* < 0.001. G: HepG2 cells (1 × 10^7^ in 100 μl DMEM) were injected subcutaneously on both upper thighs of 4-week-old male BALB/c nude mice. After 10 days, tumor-bearing mice were randomly divided into Ctrl/STA9090/NB treated group. Mice with xenograft tumor were treated with STA9090 (25 mg/kg) or NB (50 mg/kg) three times a week for 10 days. The appearance of exfoliated tumors (n = 4 per group) was shown. H: Tumor sizes were measured every 2 days, and growth curves of xenograft tumors were plotted. Data are shown as means ± SD, n = 4 mice per group, ∗*P* < 0.05, ∗∗*P* < 0.01. I: Quantification of mice weight. Data are shown as means ± SD, n = 4 mice per group, ∗*P* < 0.05. J: Heatmap analysis of 32 fatty acid species in tumors treated with STA9090 (25 mg/kg) or NB (50 mg/kg) was shown. (K) After Nile red staining of frozen section of xenograft tumors, fluorescence images were captured, and intensity quantification were analyzed. Scale bar: 100 μm. Data are shown as means ± SD, n = 4 per group, ∗∗∗*P* < 0.001. L: The protein level of FASN in xenograft tumors was detected by IHC staining. Scale bar: 100 μm. Data are shown as means ± SD, n = 3 per group, ∗*P* < 0.05. FASN, fatty acid synthetase; HCC, hepatocellular carcinoma; Hsp90, heat shock protein 90; IHC, immunohistochemistry; NB, novobiocin; TG, triglyceride.

To confirm the role of Hsp90 in lipid synthesis of HCC, we further verified the effect of Hsp90 inhibitors on lipid synthesis in HCC cell lines. Consistently, the Gene Ontology enrichment analysis in both RNA-seq and proteomics data of HepG2 cells treated with Hsp90 inhibitors demonstrated that Hsp90 suppression caused genetic changes in cellular lipid metabolism unfolded protein and protein binding (Fig. 2A, B). Next, our targeted lipidomics results of HepG2 cells showed that 41 FA species including palmitic acid (C16:0, the synthetic product of FASN) were downregulated by STA9090 or NB (Fig. 2C). Moreover, both the laser confocal microscopy images (Fig. 2D), flow cytometry analysis (Fig. 2E) of HepG2 and Huh7 cells with NR staining and TG detection (Fig. 2F) confirmed that Hsp90 inhibitors decreased the lipid accumulation. In summary, these results suggested that Hsp90 inhibitors reduced the lipid accumulation in HCC cell lines.

Next, we conducted subcutaneous tumor xenotransplantation experiments with HepG2 cells in BALB/c nude mice and treated with Hsp90 inhibitors. The average of xenografted tumor sizes in STA9090 and NB treatment group were smaller compared with control (Fig. 2G). Interestingly, Hsp90 inhibitors treatment decreased not only the growth of xenograft tumors, but also the body weights of nude mice (Fig. 2H, I). Consistent with the above results in vitro (Fig. 2C), targeted lipidomics results of xenografted tumors also revealed that C16:0 was reduced after STA9090 or NB treatment (Fig. 2J). The NR staining of frozen sections of tumors showed that after treatment with the Hsp90 inhibitors, lipid accumulation in xenografted tumors was reduced (Fig. 2K). To further explore whether FASN is the client protein of Hsp90, the BioGRID protein interaction database (https://thebiogrid.org/) was accessed, and the data suggested that FASN interacts with Hsp90α. Since the Hsp90 client protein might degrade with the treatment of Hsp90 inhibitors, IHC detection of FASN in tumor xenograft samples was performed, and the results revealed that tumors treated with STA9090, or NB led to the reductions of FASN protein levels (Fig. 2L).

### The expression level of Hsp90α and FASN are upregulated in tumor tissue samples of HCC patients and associated with poor prognosis

**Fig. 3:**
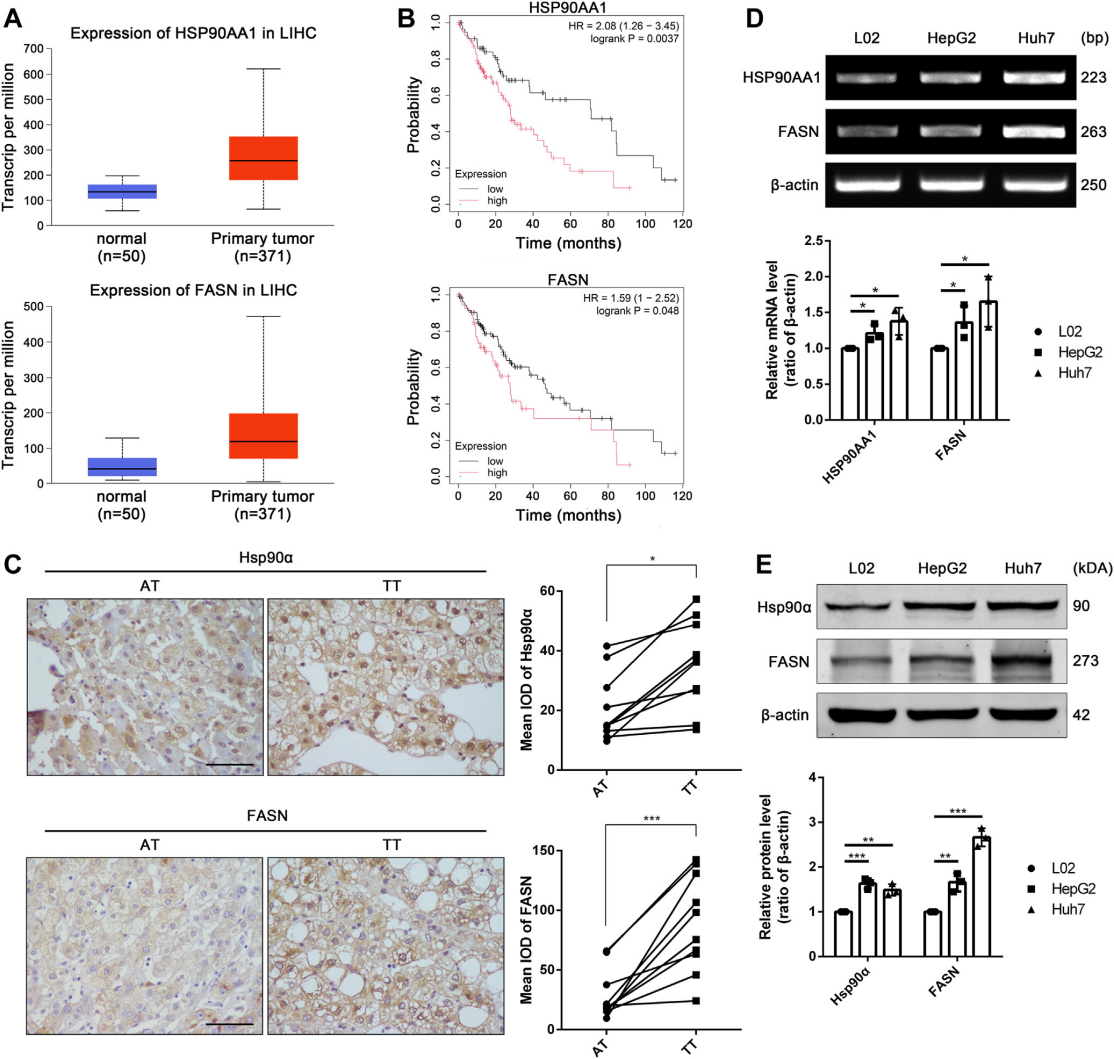
Hsp90α and FASN were upregulated in tumor tissue samples of HCC patients and associated with poor prognosis. A: TCGA data of transcription levels of Hsp90α and FASN in HCC. B: Kaplan-Meier survival curves of HCC patients (Hepatitis virus: none) based on Hsp90α and FASN protein level. C: The protein levels of Hsp90α and FASN in adjacent tissue (AT) and tumor tissue (TT) of HCC patients detected by IHC staining. Scale bar: 100 μm. Data are shown as means ± SD, n = 10 patients per group, ∗*P* < 0.05, ∗∗∗*P* < 0.001. D: The mRNA levels of Hsp90α and FASN in HCC cell lines (HepG2, Huh7) compared to hepatocyte cell line (L02) were detected by PCR. Data are shown as means ± SD, n = 3 per group, ∗*P* < 0.05. E: Protein levels of Hsp90α and FASN were detected by immunoblotting in different cells. Data are shown as means ± SD, n = 3 per group, ∗∗*P* < 0.01, ∗∗∗*P* < 0.001. FASN, fatty acid synthetase; HCC, hepatocellular carcinoma; Hsp90, heat shock protein 90; IHC, immunohistochemistry.

Next, the expression levels of Hsp90α and FASN in tumor samples derived from HCC patients were explored. The UALCAN database (http://ualcan.path.uab.edu/index.html) demonstrated that comparing with the adjacent normal liver tissue, mRNA levels of Hsp90α and FASN in HCC tumor tissue (TT) were highly expressed (Fig. 3A). In addition, the data acquired from Kaplan-Meier Plotter (https://kmplot.com/analysis/index.php?p=background) revealed that high mRNA levels of Hsp90α and FASN in liver cancer were associated with poor prognosis of patients (Fig. 3B). Our immunohistochemistry staining of 10 paired HCC TTs and comparable adjacent tissues showed that the protein levels of Hsp90α and FASN in TT from HCC patients were both higher than those in adjacent tissues (Fig. 3C). Besides, in vitro experiments were also applied to assess diversities in the expression of Hsp90 and FASN in different cells. Consistently, HCC cell lines (HepG2 and Huh7) had higher expression levels of Hsp90α and FASN in either mRNA (Fig. 3D) or proteins (Fig. 3E) when compared with normal hepatocytes LO2. The above results demonstrated that there are high expression levels of Hsp90α and FASN in HCC, associated with poor prognosis.

### NTD and CTD of Hsp90α are necessary for lipid accumulation via FASN

**Fig. 4:**
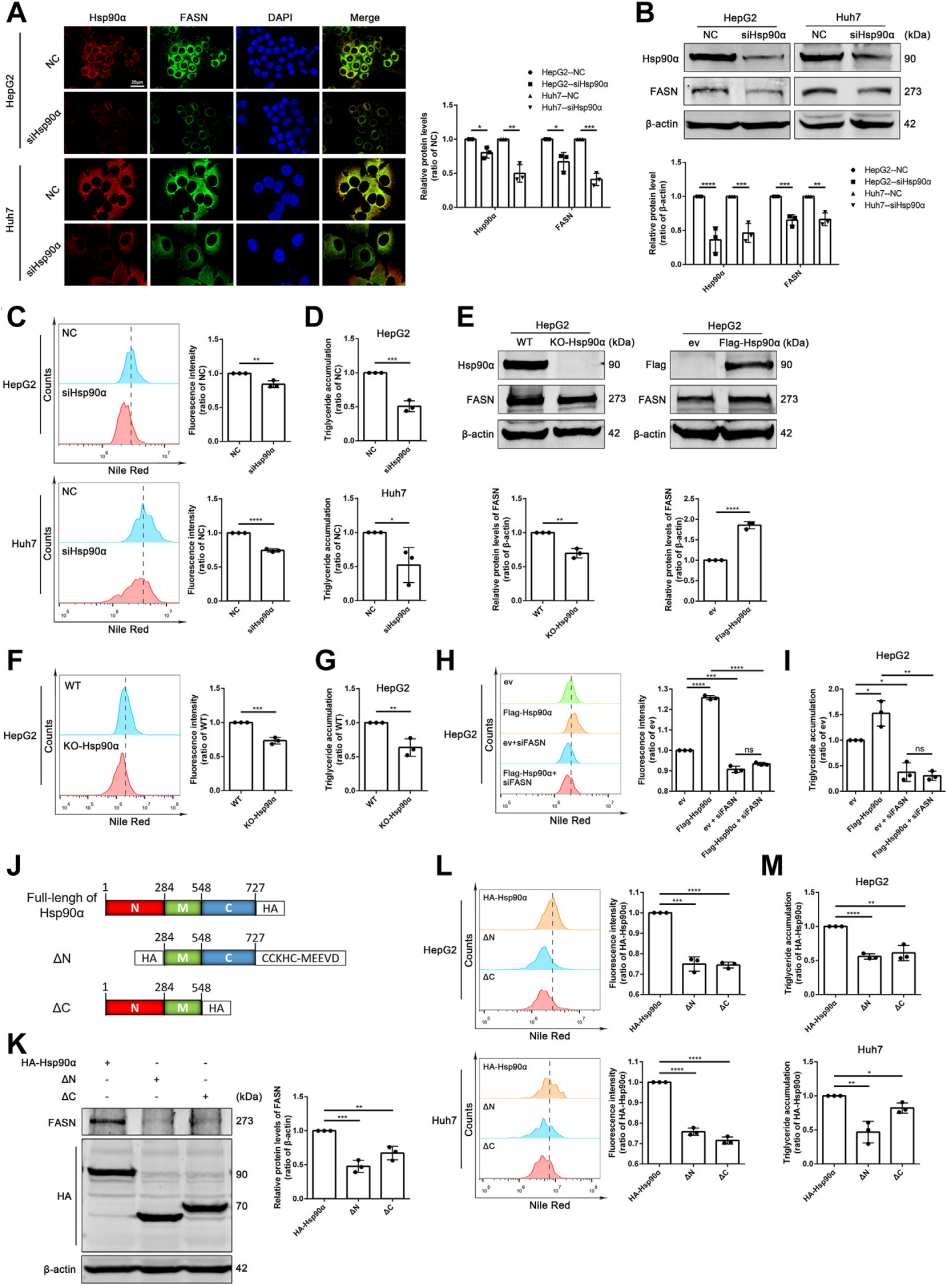
N-terminal and C-terminal domain of Hsp90α are necessary for lipid accumulation via FASN. A: Immunofluorescence staining images of Hsp90α (*red*) and FASN (*green*) in HepG2 cells transfected with siRNA targeting Hsp90α. Scale bar: 20 μm. Data are shown as means ± SD, n = 3 per group, ∗*P* < 0.05, ∗∗*P* < 0.01, ∗∗∗*P* < 0.001. B: The protein level of FASN in HepG2 and Huh7 cells after interference with Hsp90α was detected by Western blotting. Data are shown as means ± SD, n = 3 per group, ∗∗*P* < 0.01, ∗∗∗*P* < 0.001, ∗∗∗∗*P* < 0.0001. Lipid accumulation of HCC cells indicated by Nile red staining after interference with Hsp90α was analyzed by flow cytometry (C) and another lipid accumulation index, cellular TG levels were detected (D). Scale bar: 20 μm. Data are shown as means ± SD, n = 3 per group, ∗*P* < 0.05, ∗∗*P* < 0.01, ∗∗∗*P* < 0.001, ∗∗∗∗*P* < 0.0001. E: Change of FASN protein levels in HepG2 cells after Hsp90α KO or overexpression. Data are shown as means ± SD, n = 3 per group, ∗*P* < 0.05, ∗∗*P* < 0.01, ∗∗∗∗*P* < 0.0001. F and G: Lipid accumulation of HCC cells after Hsp90α KO were detected. Scale bar: 20 μm. Data are shown as means ± SD, n = 3 per group, ∗∗*P* < 0.01, ∗∗∗*P* < 0.001. H and I: Lipid accumulation of HepG2 cells after overexpression of Hsp90α combining with or without interference with FASN were detected. Scale bar: 20 μm. Data are shown as means ± SD, n = 3 per group, ∗*P* < 0.05, ∗∗*P* < 0.01, ∗∗∗*P* < 0.001, ∗∗∗∗*P* < 0.0001. J: Schematic diagram of plasmids encoding full-length Hsp90α, N-terminal deletion of Hsp90α, and C-terminal deletion of Hsp90α. K: FASN protein levels were compared in HepG2 cells after transfection with indicated Hsp90a plasmids. Data are shown as means ± SD, n = 3 per group, ∗∗*P* < 0.01, ∗∗∗*P* < 0.001, significantly different from HA-Hsp90α group. L and M: Lipid accumulation of HepG2 cells after transfection with indicated Hsp90a plasmids were detected. Scale bar: 20 μm. Data are shown as means ± SD, n = 3 per group, ∗*P* < 0.05, ∗∗*P* < 0.01, ∗∗∗*P* < 0.001, ∗∗∗∗*P* < 0.0001. FASN, fatty acid synthetase; HCC, hepatocellular carcinoma; Hsp90, heat shock protein 90; TG, triglyceride.

To test whether Hsp90α affect lipid accumulation via FASN, we analyzed the effect of Hsp90α on FASN with siHsp90α. Confocal microscopy images demonstrated the fluorescence intensity of FASN decreased after knockdown of Hsp90α (Fig. 4A). In addition, decreased protein level of FASN was also observed in Hsp90α knockdown HCC cell lines (Fig. 4B). Both confocal microscopy images (supplemental Fig. S1A) and flow cytometry results (Fig. 4C) of NR staining showed that after knocking down Hsp90α, the LD amount decreased. Consistently, the other index of lipid accumulation, the intracellular TG amounts were also downregulated in HepG2 and Huh7 cells with Hsp90α knocked down (Fig. 4D). Interestingly, we also found that Hsp90β knockdown reduced the lipid accumulation in HCC cells (supplemental Fig. S2A–C). Furthermore, KO of Hsp90α by CRISPR-cas9 with sgRNA dramatically led to a downregulation of FASN, and overexpression of Hsp90α increased the FASN protein level (Fig. 4E), indicating FASN was a candidate client protein of Hsp90α. Moreover, lipid accumulation in Hsp90α-KO HepG2 cells was decreased (Fig. 4F, G and supplemental Fig. S1B). To verify whether Hsp90α regulates lipid accumulation via FASN, we overexpressed Hsp90α combining with or without siRNA of FASN, and found that knockdown of FASN abolished the elevated lipid accumulation induced by Hsp90α overexpression in HepG2 cells (Fig. 4H, I and supplemental Fig. S1C).

To further confirm the effects of NTD and CTD of Hsp90α on FASN related lipid accumulation, we transfected HCC cells with plasmids with full length of Hsp90α (HA-tagged full-length), Hsp90α NTD deletion (HA-tagged MD-CTD) or CTD deletion (HA-tagged NBD-MD) (Fig. 4J). As is the case with STA9090 or NB treatment, NTD or CTD deletion of Hsp90α reduced FASN (Fig. 4K) and lipid accumulation (Fig. 4L, M and supplemental Fig. S1D) in HCC cells.

### Hsp90α-FASN interaction maintains FASN stability

**Fig. 5:**
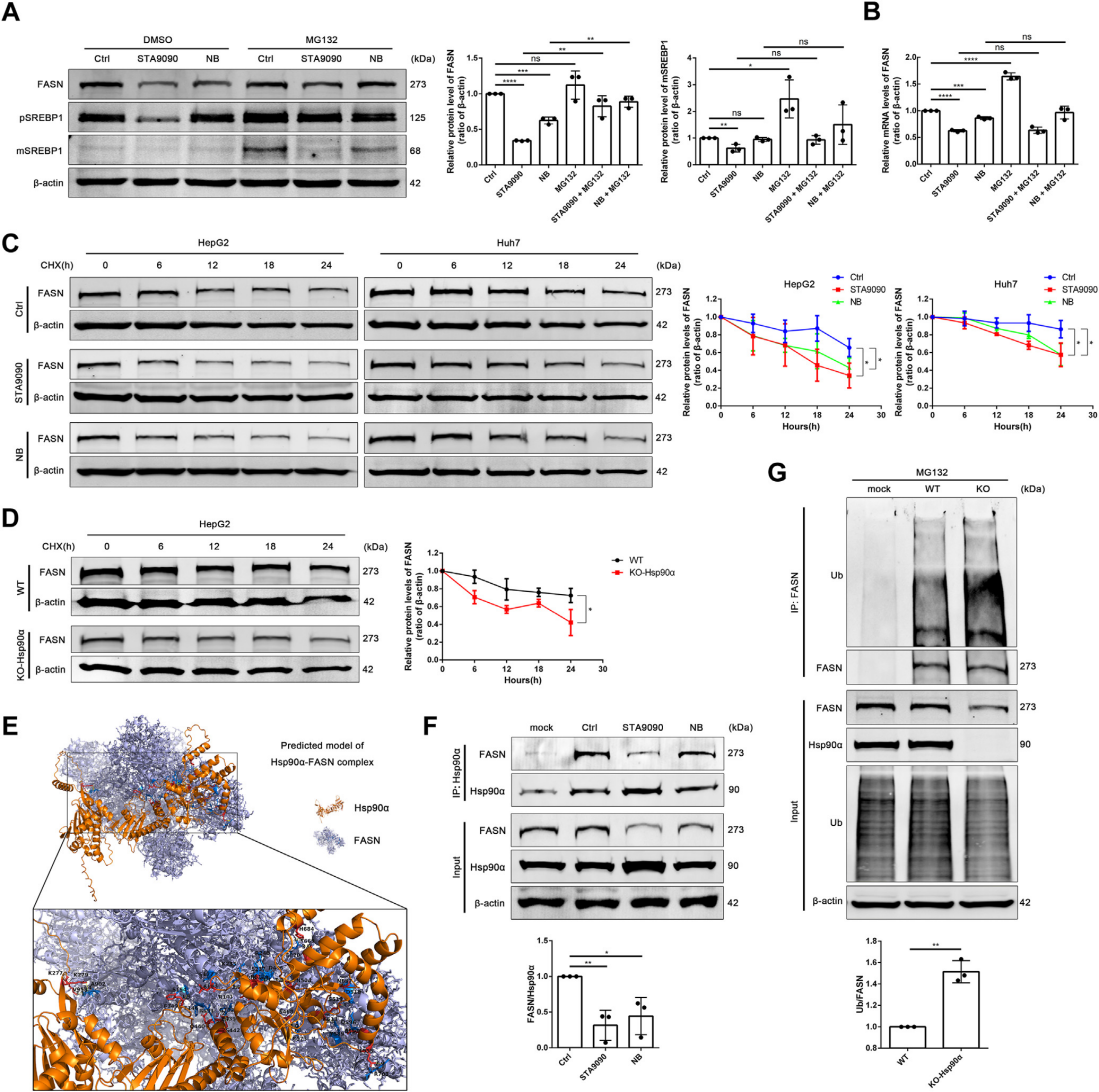
Hsp90α-FASN interaction maintains FASN stability. A: HepG2 cells were treated with DMSO (1 μl DMSO in 1 ml DMEM for 24 h) or STA9090 (100 nM for 24 h) or NB (500 μM for 24 h) combined with DMSO (1 μl/ml for 6 h) or MG132 (10 μM for 6 h). The protein levels of FASN and SREBP1 were detected by Western blotting. Data are shown as means ± SD, n = 3 per group, ∗∗*P* < 0.01, ∗∗∗*P* < 0.001, ∗∗∗∗*P* < 0.0001. B: qPCR analysis of FASN expression in HepG2 cells treated with STA9090 (100 nM for 24 h), NB (500 μM for 24 h) combined with DMSO (1 μl/ml for 6 h) or MG132 (10 μM for 6 h). Data are shown as means ± SD, n = 3 per group, ∗∗∗*P* < 0.001, ∗∗∗∗*P* < 0.0001. C: Protein half-life assay of FASN in HepG2 and Huh7 cells treated with STA9090 (100 nM) or NB (500 μM) combined with 25 μg/ml CHX for indicated times. Data are shown as means ± SD, n = 3 per group, ∗*P* < 0.05. D: Protein half-life assay of FASN in WT HepG2 and Hsp90α-KO HepG2 cells treated with 25 μg/ml CHX for indicated times. Data are shown as means ± SD, n = 3 per group, ∗*P* < 0.05. E: Molecular docking prediction model of Hsp90α and FASN. F: Interaction between Hsp90α and FASN in HepG2 cells treated with DMSO (1 μl DMSO in 1 ml DMEM for 24 h) or STA9090 (100 nM for 24 h) or NB (500 μM for 24 h) combined with MG132 (10 μM for 6 h) detected by Co-IP. Data are shown as means ± SD, n = 3 per group, ∗*P* < 0.05, ∗∗*P* < 0.01. G: Ubiquitination assay of FASN in WT HepG2 cells and Hsp90α-KO HepG2 cells treated with MG132 (10 μM for 6 h). Data are shown as means ± SD, n = 3 per group, ∗∗*P* < 0.01. Co-IP, co-immunoprecipitation; CHX, cycloheximide; FASN, fatty acid synthetase; Hsp90, heat shock protein 90; NB, novobiocin; qPCR, quantitative PCR; SREBP1, sterol regulatory element binding protein 1.

Since more than 80% of the protein in mammalian cells degrades through the 26S proteasome (37), we hypothesized that 26S proteasomal degradation was responsible for the downregulation of FASN caused by Hsp90 inhibitors. The decreasing protein level of FASN in Hsp90 inhibitors treated HepG2 cells could be partially reversed by MG132, a 26S protostome inhibitor (Fig. 5A). Additionally, we also detected the mRNA level changes of FASN affected by the above treatments and found the mRNA levels of FASN were downregulated after Hsp90 inhibitors treatment but could not be rescued by MG132 (Fig. 5B). The above results indicated Hsp90 inhibition reduced the FASN protein stability as well as the mRNA transcription. To further explore the effect of Hsp90 inhibition on FASN mRNA transcription, we first focused on SREBP1, a well-known transcription regulator of FASN. SREBP1 is also regarded as an Hsp90 client protein, since Hsp90 participates in maintaining the protein stability of SREBP1 (26). SREBP1 has a form of precursor (pSREBP1) as well as another form of mature body (mSREBP1) which plays the role of transcriptional activation. In this study, STA9090 decreased pSREBP1 protein level dramatically, and Hsp90 inhibitors reduced pSREBP1 protein level could all be partially recovered by MG132. These results indicated that STA9090, as an Hsp90 N-terminal inhibitor, may have a special function on maintaining the pSREBP1 protein stability, comparing to Hsp90 C-terminal inhibitor NB. Furthermore, the reduction of mSREBP1 under STA9090 treatment was failed to be restored by MG132 (Fig. 5A), indicating STA9090 also affected the transition process from pSREBP1 to mSREBP1. Next, to further confirm whether Hsp90 is necessary to maintain FASN protein stability, CHX was adopted to inhibit protein translation process, and we found that Hsp90 inhibitors accelerated the decay rates of FASN protein level in HCC cell lines (Fig. 5C). Consistently, the comparison between WT HepG2 cells and Hsp90α-KO HepG2 cells also showed that the KO of Hsp90α led to the increased degradation of FASN protein (Fig. 5D). Besides, the enhanced immunofluorescence colocalization between ubiquitin (Ub) and FASN after treatment with Hsp90 inhibitors suggested that inhibition of Hsp90 promote ubiquitination modification of FASN protein (supplemental Fig. S3A).

Given that Hsp90 has been reported to interact with its substrate proteins and maintain their stability, we speculated that Hsp90 might physically interact with FASN. After searching the BioGRID database (https://thebiogrid.org/), we found that Hsp90α was on the list of FASN interactors, but no more confirmative experiment was done before. Then we did immunofluorescence staining and immunoprecipitation experiment, respectively, to verify the effect of Hsp90 inhibitor on Hsp90α-FASN interaction. The confocal microscopy images showed that there was colocalization between Hsp90α and FASN, which could be suppressed by Hsp90 inhibitors (supplemental Fig. S3B). Additionally, the higher colocalization level between Ub and FASN was also found in Hsp90α-KO HepG2 cells than in WT HepG2 cells (supplemental Fig. S3C). The protein data of Hsp90α and FASN obtained from AlphaFold (https://alphafold.ebi.ac.uk/) were imported into Vakser GRAMM (https://gramm.compbio.ku.edu/) for protein molecular docking. The predicted result of the complex model showed that both the NTD and CTD of Hsp90α could interact with FASN (Fig. 5E). Consistently, Co-IP further proved that Hsp90α interacted with FASN, and Hsp90 inhibitors reduced the interaction (Fig. 5F). Furthermore, Co-IP also verified that the ubiquitination level of FASN in HepG2 cells with Hsp90α KO was higher compared with WT HepG2 cells (Fig. 5G). Overall, these data suggested that Hsp90α interacted with FASN and suppressed the ubiquitin-proteasomal degradation of FASN.

### Inhibition of the NTD of Hsp90α reduces nucleus location of SREBP1 and Hsp90 inhibition decreases the binding of SREBP1 on FASN promoter

**Fig. 6:**
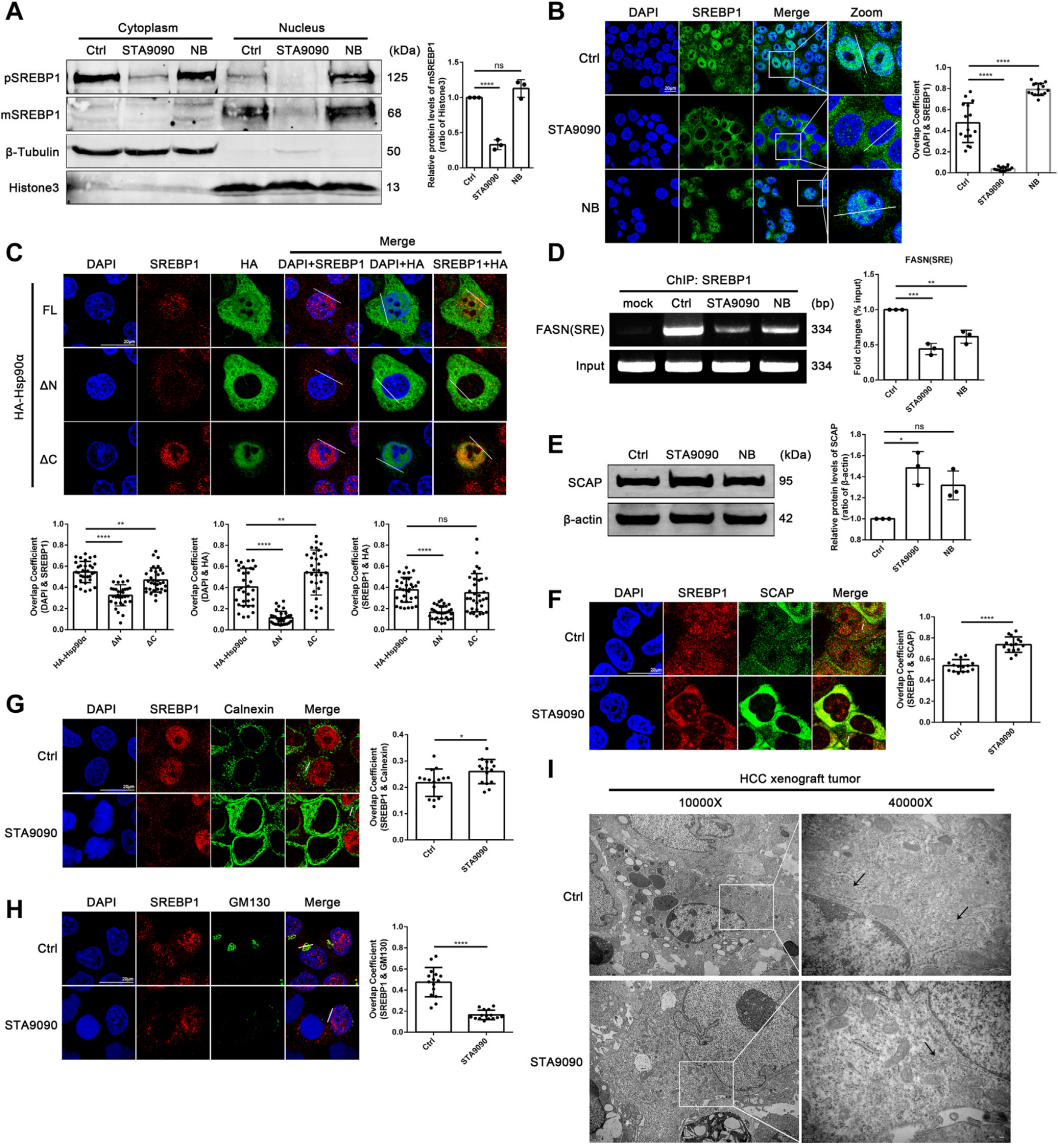
The N-terminal domain of Hsp90α is essential for SREBP1 to activate transcription of FASN. A: Cytoplasmic and nuclear distribution of SREBP1 in HepG2 cells treated with DMSO (1 μl DMSO in 1 ml DMEM for 24 h) or STA9090 (100 nM for 24 h) or NB (500 μM for 24 h). Data are shown as means ± SD, n = 3 per group, ∗∗∗∗*P* < 0.0001. B: Immunofluorescence staining for SREBP1 (*green*) and DAPI (*blue*) was performed in HepG2 cells treated with DMSO (1 μl DMSO in 1 ml DMEM for 24 h) or STA9090 (100 nM for 24 h) or NB (500 μM for 24 h). Scale bar: 20 μm. Data are shown as means ± SD, n = 15 cells per group, ∗∗∗∗*P* < 0.0001. C: Immunofluorescence staining for SREBP1 (*red*), HA (*green*) and DAPI (*blue*) was performed in HepG2 cells transfected with indicated Hsp90α plasmids. Scale bar: 20 μm. Data are shown as means ± SD, n = 32 cells per group, ∗∗*P* < 0.01, ∗∗∗∗*P* < 0.0001. D: ChIP analysis of SREBP1 binding to the SRE of *FASN* promoter in HepG2 cells treated with DMSO (1 μl DMSO in 1 ml DMEM for 24 h) or STA9090 (100 nM for 24 h) or NB (500 μM for 24 h). PCR was performed with primers specific to the SREBP1-binding motifs. Results were normalized to the input. Data are shown as means ± SD, n = 3 per group, ∗∗*P* < 0.01, ∗∗∗*P* < 0.001. E: HepG2 cells were treated with DMSO (1 μl DMSO in 1 ml DMEM for 24 h) or STA9090 (100 nM for 24 h) or NB (500 μM for 24 h). The protein levels of SCAP were detected by Western blotting. Data are shown as means ± SD, n = 3 per group, ∗*P* < 0.05. F: Immunofluorescence staining for SREBP1 (*red*) and SCAP (*green*) was performed in HepG2 cells treated with DMSO (1 μl DMSO in 1 ml DMEM for 24 h) or STA9090 (100 nM for 24 h) or NB (500 μM for 24 h). Scale bar: 20 μm. Data are shown as means ± SD, n = 15 cells per group, ∗∗∗∗*P* < 0.0001. G: Immunofluorescence staining for SREBP1 (*red*) and Calnexin (*green*) was performed in HepG2 cells treated with DMSO (1 μl DMSO in 1 ml DMEM for 24 h) or STA9090 (100 nM for 24 h) or NB (500 μM for 24 h). Scale bar: 20 μm. Data are shown as means ± SD, n = 15 cells per group, ∗*P* < 0.05. H: Immunofluorescence staining for SREBP1 (*red*) and GM130 (*green*) was performed in HepG2 cells treated with DMSO (1 μl DMSO in 1 ml DMEM for 24 h) or STA9090 (100 nM for 24 h) or NB (500 μM for 24 h). Scale bar: 20 μm. Data are shown as means ± SD, n = 15 cells per group, ∗∗∗∗*P* < 0.0001. I: Transmission electron microscope images of HCC xenograft tumors treated with STA9090 (25 mg/kg), Scale bar: 2 μm or 500 nm indicated in the figures. Black arrows point to the Golgi apparatus. ChIP, chromatin immunoprecipitation; DAPI, 4',6-diamidino-2-phenylindole; FASN, fatty acid synthetase; HCC, hepatocellular carcinoma; Hsp90, heat shock protein 90; NB, novobiocin; SREBP1, sterol regulatory element binding protein 1.

We next focused on the mechanism of the influences of different Hsp90 inhibitors on SREBP1. We isolated cytoplasmic and nuclear proteins in HepG2 cells and found that the N-terminal inhibitor of Hsp90 reduced SREBP1 level and restrained the location of SREBP1 in nucleus (Fig. 6A). It was further confirmed by the immunofluorescence colocalization between SREBP1 and the nucleus (4',6-diamidino-2-phenylindole), to show the nucleus localization of SREBP1. Interestingly, STA9090 treatment abolished the nucleus localization of Hsp90α and significantly decreased SREBP1 nucleus localization (Fig. 6B). Similarly, transfection of Hsp90α NTD deletion plasmid in HepG2 cells caused a decline of the nuclear localization of SREBP1 (Fig. 6C). These results strongly supported that Hsp90α NTD is necessary for the nucleus localization of Hsp90α which is related to SREBP1 amount in nucleus. Furthermore, the results of ChIP-PCR revealed that the binding between SREBP1 and *FASN* promoter was decreased by Hsp90 inhibitors, especially after the treatment of STA9090, which showed a dramatical reduction (Fig. 6D) and was consistent with the significant decrease in SREBP1 level in the nucleus of STA9090 treated HepG2 cells.

Given that the nucleus translocation of SREBP1 is vital for its transcription activity, and SREBP cleavage-activating protein (SCAP) regulates the nuclear import SREBP1 (38). In conditions of high sterol levels, SCAP-SREBP1 interaction keeps SREBP1 (pSREBP1) in the endoplasmic reticulum (ER), preventing SREBP1 from moving to the Golgi apparatus. When sterol levels are low, SCAP releases SREBP1 from the ER to the Golgi apparatus, where SREBP1 is proteolytically cleaved. This processing releases the active N-terminal fragment of SREBP1 (mSREBP1), which is the mature form capable of entering the nucleus, where it acts as a transcription factor, enhancing the targeted gene transcription like *FASN*.

Since STA9090 obviously decreased SREBP1 nuclear location, we next tested the effect of Hsp90 inhibitors on SCAP and found an increase protein level of SCAP under STA9090 treatment (Fig. 6E). Immunofluorescence colocalization between SREBP1 and SCAP was showed to be enhanced by STA9090 (Fig. 6F). In addition, the colocalization between SREBP1 and Calnexin (ER marker) was consistently enhanced after STA9090 treatment (Fig. 6G), while the fluorescence intensity of the GM130 (Golgi marker) was significantly decreased and more fragmented, accompanied by weakened colocalization between SREBP1 and GM130 (Fig. 6H). As the processing of SREBP1 in the Golgi apparatus is a key step before its entry into the nucleus, we hypothesized that STA9090 jeopardized the function of Golgi apparatus. The transmission electron microscopy images captured from xenografted tumors revealed abnormal structure and smaller size of Golgi in STA9090-treated group (Fig. 6I). Our findings indicated inhibition of the NTD of Hsp90α downregulated nucleus location of SREBP1 and Hsp90 inhibition decreased the binding of SREBP1 on FASN promoter.

### Hsp90α regulates FASN transcription via LXRα/SREBP1 axis

**Fig. 7:**
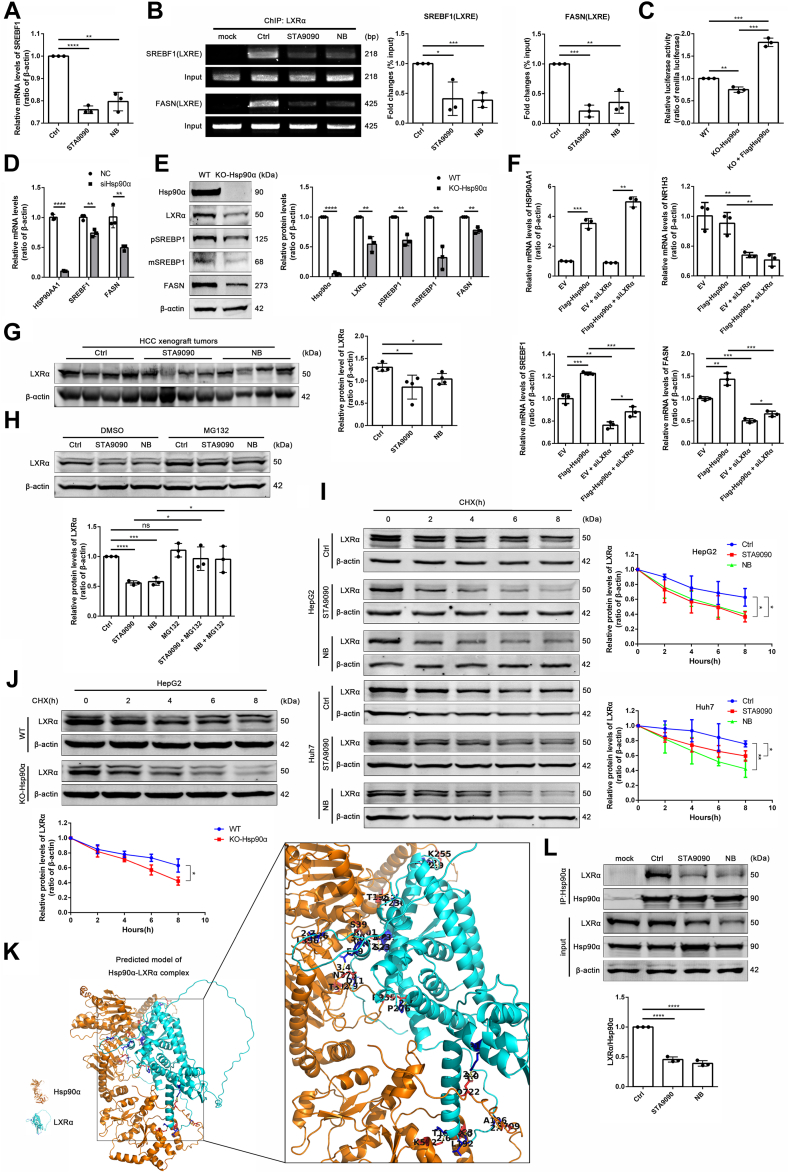
Hsp90α regulates FASN transcription via LXRα/SREBP1 axis. A: mRNA levels of SREBP1 in HepG2 cells treated with DMSO (1 μl DMSO in 1 ml DMEM for 24 h) or STA9090 (100 nM for 24 h) or NB (500 μM for 24 h) were detected by qPCR. Data are shown as means ± SD, n = 3 per group, ∗∗*P* < 0.01, ∗∗∗∗*P* < 0.0001. B: ChIP analysis of LXRα binding to the LXRE of *SREBF1* and *FASN* promoters in HepG2 cells treated with DMSO (1 μl DMSO in 1 ml DMEM for 24 h) or STA9090 (100 nM for 24 h) or NB (500 μM for 24 h). PCR was performed with primers specific to the LXRα-binding motifs. Results were normalized to the input. Data are shown as means ± SD, n = 3 per group, ∗*P* < 0.05, ∗∗*P* < 0.01, ∗∗∗*P* < 0.001. C: HepG2 cells (WT/KO-Hsp90α) were cotransfected with LXRE luciferase reporter vector plasmid and renilla luciferase plasmid combined with or without overexpression of Hsp90α. Dual-luciferase reporter gene assays detected the transcriptional activation in the LXRE region of promoter. Results were normalized to the renilla luciferase activity. Data are shown as means ± SD, n = 3 per group, ∗∗*P* < 0.01, ∗∗∗*P* < 0.001. D: mRNA levels of *HSP90AA1*, *SREBF1*, and *FASN* in HepG2 cells transfected with siRNA of *HSP90AA1* were detected by qPCR. Data are shown as means ± SD, n = 3 per group, ∗∗*P* < 0.01, ∗∗∗∗*P* < 0.0001. E: Protein levels of Hsp90α, LXRα, pSREBP1, mSREBP1, and FASN in Hsp90α-KO HepG2 cells were detected by Western blotting. Data are shown as means ± SD, n = 3 per group, ∗∗*P* < 0.01, ∗∗∗∗*P* < 0.0001. F: mRNA levels of *HSP90AA1*, *NR1H3*, *SREBF1*, and *FASN* in HepG2 cells after overexpression of Hsp90α combined with or without siLXRα were detected by qPCR. Data are shown as means ± SD, n = 3 per group, ∗*P* < 0.05, ∗∗*P* < 0.01, ∗∗∗*P* < 0.001. G: Protein levels of LXRα in HCC xenograft tumors treated with STA9090 (25 mg/kg) or NB (50 mg/kg) were detected by Western blotting. Data are shown as means ± SD, n = 4 per group, ∗*P* < 0.05. H: HepG2 cells were treated with DMSO (1 μl DMSO in 1 ml DMEM for 24 h) or STA9090 (100 nM for 24 h) or NB (500 μM for 24 h) combined with DMSO (1 μl/ml for 6 h) or MG132 (10 μM for 6 h). The protein levels of LXRα were detected by Western blotting. Data are shown as means ± SD, n = 3 per group, ∗*P* < 0.05, ∗∗∗*P* < 0.001, ∗∗∗∗*P* < 0.0001. I: Protein half-life assay of LXRα in HepG2 and Huh7 cells treated with STA9090 or NB combined with 25 μg/ml CHX for indicated times. Data are shown as means ± SD, n = 3 per group, ∗*P* < 0.05, ∗∗*P* < 0.01. (J) Protein half-life assay of LXRα in WT HepG2 and Hsp90α-KO HepG2 cells treated with 25 μg/ml CHX for indicated times. Data are shown as means ± SD, n = 3 per group, ∗*P* < 0.05. K: Molecular docking prediction model of Hsp90α and LXRα. L: Interaction between Hsp90α and LXRα in HepG2 cells treated with STA9090, NB detected by co-IP. Data are shown as means ± SD, n = 3 per group, ∗∗∗∗*P* < 0.0001. Co-IP, co-immunoprecipitation; ChIP, chromatin immunoprecipitation; CHX, cycloheximide; FASN, fatty acid synthetase; HCC, hepatocellular carcinom; Hsp90, heat shock protein 90; LXR, liver X receptor; mSREBP1, mature SREBP1; NB, novobiocin; pSREBP1, precursor SREBP1; qPCR, quantitative PCR; SREBP1, sterol regulatory element binding protein 1.

Since decreased SREBP1 protein level caused by Hsp90 inhibitors treatments could only be partially reversed by MG132, we next explored whether Hsp90 inhibitors reduced transcripts of SREBP1 in HCC cells. Interestingly, Hsp90 inhibitors suppressed SREBP1 at the transcriptional level (Fig. 7A). It is known that LXR as a transcription factor regulating genes of de novo FA synthesis, including *SREBF1*, *FASN* are closely related to FA synthesis required for cell proliferation (12). We hypothesized Hsp90 inhibitors also might downregulate mRNA expression of SREBP1 and FASN via LXRα in HCC. Thus, we investigated the combinations of LXRα with the specific binding element LXRE on the *SREBF1* promoter and the *FASN* promoter through ChIP-PCR and found that Hsp90 inhibitors decreased the binding of LXRα at the promoter of *SREBF1* and *FASN* (Fig. 7B). In addition, reporter gene assay demonstrated that Hsp90α KO reduced the transcriptional activity of LXRE in HepG2 cells, while overexpression of Hsp90α had an opposite effect (Fig. 7C). Consistently, Hsp90α-knockdown resulted in lower mRNA levels of SREBP1 and FASN (Fig. 7D), while the protein levels of LXRα, SREBP1, and FASN were downregulated in Hsp90α-KO HepG2 cell line (Fig. 7E). To investigate whether the effect of Hsp90α occurs in the absence of LXRα, transcription levels of SREBP1 and FASN in HepG2 cells after overexpression of Hsp90α combined with LXRα knockdown were detected. The mRNA of both SREBP1 and FASN were downregulated after LXRα knockdown but partially recovered when combined with overexpression of Hsp90α (Fig. 7F). Moreover, changes in lipid accumulation of HepG2 cells were consistent with the trend of SREBP1 and FASN under the same treatment (supplemental Fig. S4A–C). Interestingly, overexpression of Hsp90β combined with knockdown of LXRα showed similar effects in HepG2 cells (supplemental Fig. S5A–D). Western blot showed that LXRα in xenografted tumors were reduced by Hsp90 inhibitors (Fig. 7G). To determine whether Hsp90 inhibitors promoted LXRα degradation via proteasomal pathway, HepG2 cells were treated with Hsp90 inhibitors with or without MG132. We found that proteasome inhibitor MG132 restored the decrease of LXRα caused by Hsp90 inhibitors (Fig. 7H). Moreover, protein half-life assay demonstrated that Hsp90 inhibitors shorten the protein half-life times of LXRα in HepG2 and Huh7 cells (Fig. 7I). Consistent with this result, Hsp90α KO in HepG2 cells also led to a shorter protein half-life of FASN (Fig. 7J). Based on the BioGRID database, an affinity capture-Western blot analysis of Hsp90-target interactions revealed an interaction between Hsp90α and LXRα (39). Besides, the predicted model of the complex of Hsp90α and LXRα was obtained via Vakser GRAMM, suggested that there are potential binding sites between Hsp90α and LXRα (Fig. 7K). Co-IP assays further confirmed that LXRα was present in Hsp90α immunoprecipitates from HepG2 cells and Hsp90 inhibitors reduced the levels of LXRα pulled down from Hsp90α (Fig. 7L). Taken together, these findings suggested that Hsp90α inhibition decreased both SREBP1 and FASN transcription by promoting the degradation of LXRα and reducing the binding of LXRα to LXRE.

## Discussion

The application of anticancer drugs or other treatments were not effective enough to significantly extend the survival period of HCC patients especially for those with advanced HCC (2). Cellular metabolic reprogramming is a vital biological feature of HCC (40, 41), therefore, exploring the metabolic characteristics and underlying molecular mechanisms of HCC is critical to improve the effectiveness of HCC treatment.

In this study, we verified that HCC cells had more lipid accumulation than normal hepatocytes, accompanied with increased mRNA and protein level of FASN. As the rate-limiting enzyme of de novo FA synthesis, FASN had been found to be upregulated in various tumors and associated with biological effects such as proliferation, migration, and invasion (42, 43). Furthermore, the assemblies of a variety of lipids including TGs need supplies from FASN-related lipogenesis, and LDs mainly formed by TGs in cancer cells can act as a buffer zone to prevent ER stress (44), eliminate reactive oxygen species (45), adsorb and isolate antitumor drugs, thus enabling HCC to acquire drug resistance (46).

Hsp90 functions as a foldase with dynamic conformation, binds to various substrate proteins with its different domains, assisting in the proper folding and assembly of its substrate proteins (20, 47). Since the Hsp90 substrate proteins have multiple important roles in cancer, Hsp90 inhibition has been the candidate for therapeutic interest for more than 2 decades (48). In this study, our RNA-seq analysis indicated that under the treatment of Hsp90 inhibitors STA9090 and NB, gene expression of HepG2 cells showed changes in lipid metabolism. Besides, proteomic analysis of HepG2 cells suggested that Hsp90 inhibitors induced changes in FA metabolism and TG metabolism. Therefore, we further carried out lipidomics analysis in HepG2 cells and found that the abundance of various FAs including palmitic acid (C16:0) was downregulated by Hsp90 inhibitors. All these suggested that lipid metabolism in HCC cells is affected by Hsp90 inhibitors, possibly due to regulatory effects between Hsp90 and lipid metabolism-related enzymes that are altered after Hsp90 inhibition, especially FASN, which produces C16:0. We found that inhibition of Hsp90 caused tumor growth inhibition and weight loss in xenografted animal models, and correspondingly, FASN levels and lipid accumulation of HCC cells were downregulated in vivo and in vitro. Consistent with the results above, higher levels of Hsp90α and FASN were found in vivo and in vitro, accompanied with lower overall survival rate. These result suggested that targeting FASN-related lipid synthesis pathway via Hsp90α should be a promising treatment strategy for HCC cells to conquer drug resistance or adaptation to stressed cancer microenvironment.

Based on targeted inhibition of FASN in cancer therapy for its role in FA metabolism reprogram (49, 50), there were different studies focused on transcription, protein stability, and posttranslational modification of FASN. For instance, metformin was applied to target clusterin and inhibit lipid synthesis and the proliferation of bladder cancer cells via FASN (51). GNPAT promoted lipid synthesis and tumorigenesis in HCC via inhibiting TRIM21-mediated degradation of FASN (52). Acetylation of FASN promoted its proteasomal degradation via TRIM21 (53, 54). Promising inhibitors of FASN like Fasnall, orlistat, and TVB-3664, were developed and applied in clinical trials, but none of them are approved for cancer treatment (55). In order to better exert antitumor effects, it is of great potential significance to find more mechanisms to target FASN. Our data determined that knockdown of Hsp90α resulted in low expression of FASN and decreased lipid accumulation in HCC cells, which was verified in Hsp90α-KO HepG2 cells as well. In addition, we overexpressed Hsp90α in HCC leading an increase in lipid accumulation, and this effect was reversed when combined with knockdown of FASN. These findings revealed that FASN was a key enzyme in the upregulation of lipid synthesis by Hsp90α. Besides, transfecting with Hsp90α domain deletion plasmid (ΔN or ΔC) in HCC cells led to a decline of FASN, and a corresponding decrease in lipid accumulation was also observed, indicating that these two terminal domains were both indispensable for Hsp90α to regulate FASN-related lipid synthesis.

It is well-known that the proteasome is the main site of protein degradation in cells. The close link between Hsp90 and FASN was further explored by the protostome inhibitor MG132. We found that the reduction in FASN protein levels caused by Hsp90 inhibitors was partially restored by MG132. Moreover, although MG132 restored the level of pSREBP1, a precursor form of FASN's upstream transcription factor SREBP1, the reduction of mSREBP1 as mature form of SREBP1 induced by STA9090 was not restored. Of note, FASN mRNA levels were decreased by Hsp90 inhibitors and were not recovered by MG132. Furthermore, Hsp90 inhibitors increased the binding of Ub to FASN and reduced the protein half-life of FASN. Regulation of protein ubiquitination is associated with cellular lipid metabolism and mediates the development of HCC (56). Notably, deubiquitinases (USP2a, USP14, and USP22) that regulate the deubiquitylation and stability of FASN in various disease models had been identified (57, 58, 59). Our research determined that FASN was a substrate protein of Hsp90α, the interaction between Hsp90α and FASN stabilized FASN protein by preventing proteasomal degradation of FASN. On the other hand, Hsp90 inhibitors broke the binding between Hsp90α and FASN and promoted protein degradation of FASN through proteasome independent of SREBP1 transcriptional activation pathway. This was confirmed by ubiquitination detection in HepG2 cells, we observed that Hsp90α KO caused a higher ubiquitination level of FASN, indicating that the existence of Hsp90α was of importance for maintaining the stability of FASN. Taken together, targeting the FASN-related lipid synthesis pathway through Hsp90α may be a novel avenue in HCC therapy.

With the participation of SCAP on ER, SREBP1 is allowed to transfer to Golgi apparatus, where it is processed and releases its NTD into the nucleus to activate transcription of genes related to lipid synthesis (38). Previous studies had reported that Hsp90 interacted with SCAP-SREBP1 complex and maintained stability of the complex (27). Our studies suggested that inhibition of Hsp90α NTD prevented SREBP1 from migrating to the nucleus and activating *FASN* promoter. Interestingly, this effect seems to be attributed to the abnormal morphology of the Golgi apparatus. Since numerous proteins delivered from ER are further modified and packaged in Golgi apparatus, followed by being delivered to specific sites within the cell or extracellularly secreted. The fragmented Golgi apparatus caused by Hsp90α NTD inhibitor STA9090 may lose the ability to process SREBP1, resulting in increased anchoring of SREBP1 to SCAP in the ER. However, the mechanism of how STA9090 affects Golgi apparatus remains to be explored. Interestingly, upregulated SCAP protein levels were observed after STA9090 treatment. This increase in SCAP may be compensatory, since the downstream SREBP1-related lipid synthesis pathway was repressed and SCAP is indispensable for the translocation of SREBP1 between ER and Golgi apparatus.

Researches on LXR had emerged since the 1990s, and it is of great potential in the therapy of metabolic diseases. However, the activation of LXR promoted the transcription of liver lipid synthesis related genes like *SREBF1*, *FASN*, *SCD*, and *ACACA*, leading to liver steatosis and hyperlipidemia (12, 60). We observed that mRNA levels of SREBP1 was suppressed by Hsp90 inhibitors, so we focused on Hsp90 inhibition of the transcription factor LXRα for it plays the role of upstream transcriptional regulation of SREBP1 and FASN. Interestingly, we found that LXRα was a target of Hsp90α. Our data suggested that Hsp90α interacted with LXRα to prevent its degradation via proteasome pathway, thus ensuring the bindings of LXRα on of both *SREBF1* promoter region and *FASN* promoter region and promoting the transcription of *SREBF1* and *FASN*. Moreover, apart from Hsp90α, this study found that Hsp90β also contributed to the lipid accumulation of HCC through the LXRα/SREBP1/FASN axis, but the specific mechanism of this similar systemic effect remains to be further explored.

**Fig. 8:**
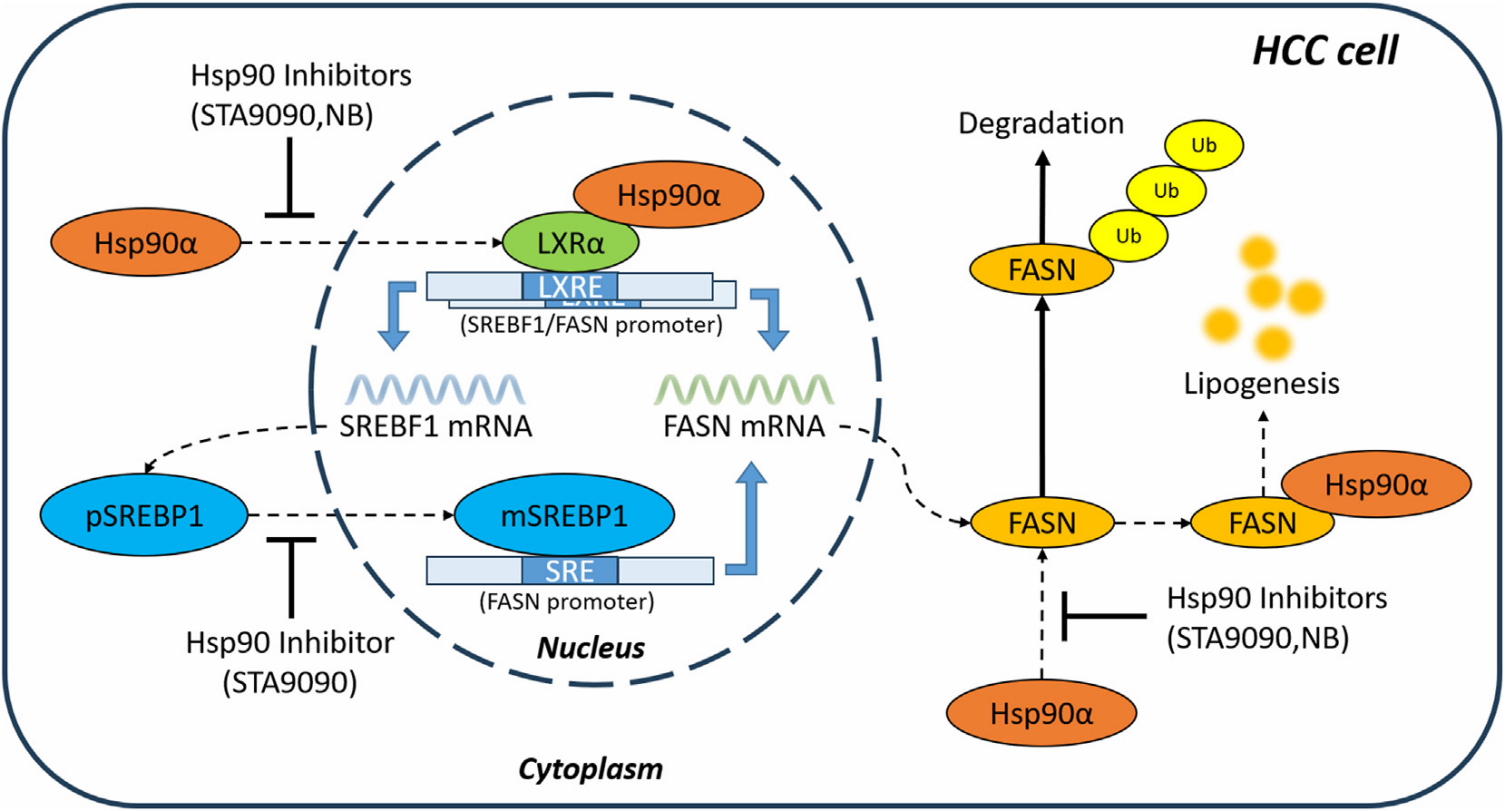
Molecular mechanism diagram of Hsp90α regulating lipid synthesis in hepatocellular carcinoma by mediating transcription and protein stability of FASN. Hsp90 inhibitors reduce the interactions between Hsp90α and FASN, as well as Hsp90α and LXRα, thereby reducing the protein stability of FASN, and downregulate the FASN from the transcriptional level via LXRα/SREBP1 axis. FASN, fatty acid synthetase; Hsp90, heat shock protein 90; SREBP1, sterol regulatory element binding protein 1; LXR, liver X receptor.

The mechanism diagram of this study showed how Hsp90α regulated lipogenesis in HCC via FASN (Fig. 8). All these data revealed a new mechanism that Hsp90α promoted lipid accumulation in HCC cells by maintaining FASN protein stability and upregulating FASN mRNA transcription through LXRα/SREBP1 axis, thus creating a favorable condition for survival and proliferation of HCC cells, providing a new strategy for the clinical treatment of HCC in lipid metabolism related pathway.

## Data availability

Data are available from the corresponding author upon reasonable request.

## Supplemental data

This article contains supplemental data (61).

## Conflict of interest

The authors declare that they have no conflicts of interest with the contents of this article.
