# Supplementary material for: Hsp90α promotes lipogenesis by stabilizing FASN and promoting FASN transcription via LXRα in hepatocellular carcinoma
Source: J Lipid Res. 2024 Dec 5;66(1):100721. doi: 10.1016/j.jlr.2024.100721 (PMC11745951; doi:10.1016/j.jlr.2024.100721)
Supplement: Supplementary Information [file mmc1.docx]

**Title:** Hsp90α promotes lipogenesis by stabilizing FASN and promoting FASN transcription via LXRα in hepatocellular carcinoma

**Contents**

1. Supplementary Material and Methods
2. Supplementary Figures 1-5
3. Supplementary Tables 1-5

**Supplementary Material and Methods**

1. **CRISPR-Cas9 plasmids (KO-HSP90α)**

|  | | |
| --- | --- | --- |
| **Catalog No.:**DC-HTN000436-D08-10 | |  |
| **Whole Plasmid Size:**9579 bp |  |  |
| **Description:**Donor clone | |  |
| **Vector:**pDonor-D08 | **Antibiotic:**Ampicillin |  |
| **Stable Selection Marker:**Neomycin | |  |
|  | | |

**Vector Information for DC-HTN000436-D08-10 (GeneCopoeia, Inc., Rockville, MD)**


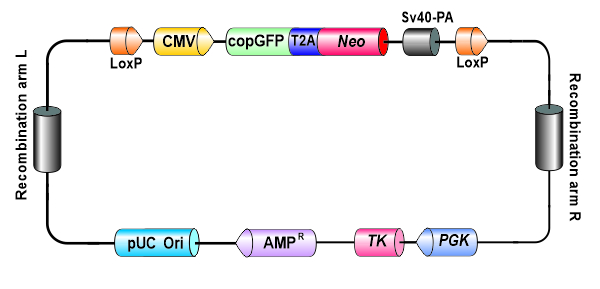


In short, single guide RNA (sgRNA) directs Cas9 endonuclease cleavage between the -3 and -4 bases upstream of the NGG sequence and the downstream of sgRNA-targeted sequence of the HSP90AA1 gene. LoxP sites are then inserted between the cleavage sites by homologous recombination.

1. **Sample preparation for mass spectrometry and Proteomics analysis**

Sample preparation was carried out as described previously [61]. HepG2 cells were lysed in the lysis buffer (8 M urea, 1 % Protease Inhibitor Cocktail) then sonicated and the supernatant was collected after centrifugation. Each sample was treated with 5 mM dithiothreitol (56°C) to be reduce and 11 mM iodoacetamide (room temperature) to be alkylate. Using trypsin to digest the proteins (37°C, overnight) at the ratio of 1:50 (w/w, trypsin-to-sample) and then at the ratio of 1:100 (w/w, trypsin-to-sample) for other 4 h. The enzymatically hydrolyzed protein peptide segments were treated with Strata X C18 (Phenomenex) for desalting, then dried by freeze-drying in vacuo. 0.5 M TEAB was added to dissolve the protein peptide segments. The tryptic peptides were separated into 60 fractions from the gradient of 8% to 32% acetonitrile (pH=9) within 1 hour Agilent 300 Extend C18 column. All fractions were vacuum-frozen dried, and prepared for liquid chromatography-mass spectrometry separation. The protein peptide segments were dissolved in solvent A (0.1% formic acid and 2% acetonitrile), and then separated on the EASY-nLC 1000 UPLC system. Solvent B (0.1% formic acid and 90% acetonitrile) was prepared, and the gradient parameters were set as follows: 7-22% solvent B (26 min); 22-32% solvent B (8 min); 32-38% solvent B (3 min); 38-80% solvent B (3 min). The protein peptide segments separated by the gradient were ionized by the NSI ion source, and the ionized protein peptide segments were analyzed by the Thermo Scientific^TM^ Orbitrap Fusion^TM^ Lumos mass spectrometer.

1. **Lipidomics mass spectrometry analysis**

A mixed standard stock solutions of fatty acids (4000 μg/mL) were diluted by the N-hexane to make the calibration curve covering a range from 1 to 2000 μg/mL (1, 5, 10, 25, 50, 100, 250, 500, 1000 and 2000 μg/mL). The samples were accurately weighed and loaded into a 2 mL centrifuge tube with 1 mL chloroform methanol (2:1) solution and 100 mg glass beads. Then the samples were put into a high-throughput tissue grinder, shaking at 55 Hz for 1 min twice and centrifuged at 12000 rpm for 5 min at 4℃ after which extracted in an ultrasonic instrument for 30 min. The supernatant was collected into a 10 mL glass centrifuge tube and fully mixed with 2 mL 1% sulfuric acid methanol solution. 1 mL n-hexane was added after esterification in a water bath at 80℃ for 30 min. Then 5 mL H_2_O (4℃) was added for washing after vortex and stand for 2 min. The samples were centrifuged at 3500 rpm for 10 min at 4℃ and 700 μL supernatant was collected into a 2 mL centrifuge tube, then 100 mg anhydrous sodium sulfate powder was added to remove excess water. 300 μL of supernatant was transferred into a 2 mL centrifuge tube after vortex. Finally 15 μL methyl salicylate (500 ppm) was added as an internal standard to the supernatant before GC-MS analysis. The GC analysis was performed on TRACE 1300 gas chromatograph (Thermo Fisher Scientific, USA). The GC was fitted with a capillary column Thermo TG-FAME (50 m*0.25 mm ID*0.20 μm) and helium was used as the carrier gas at 0.63 mL/min. Injection was made in split mode at 8:1 with an injection volume of 1 μL and an injector temperature of 250℃. The temperature of the ion source and MS transfer line were 300℃ and 280℃, respectively. The column temperature was programmed to increase from an initial temperature of 80℃, which was maintained for 1 min, followed by an increase to 160℃ at 20℃/min, which was maintained for 1.5 min, and increase to 196℃ at 3℃/min, which was maintained for 8.5 min, and finally to 250℃ at 20℃/min and kept at this temperature for 3 min. Mass spectrometric detection of metabolites was performed on TSQ 9000 (Thermo Fisher Scientific, USA) with electron impact ionization mode. Single ion monitoring (SIM) mode was used with the electron energy of 70 eV.

**Supplemental Figures**

**
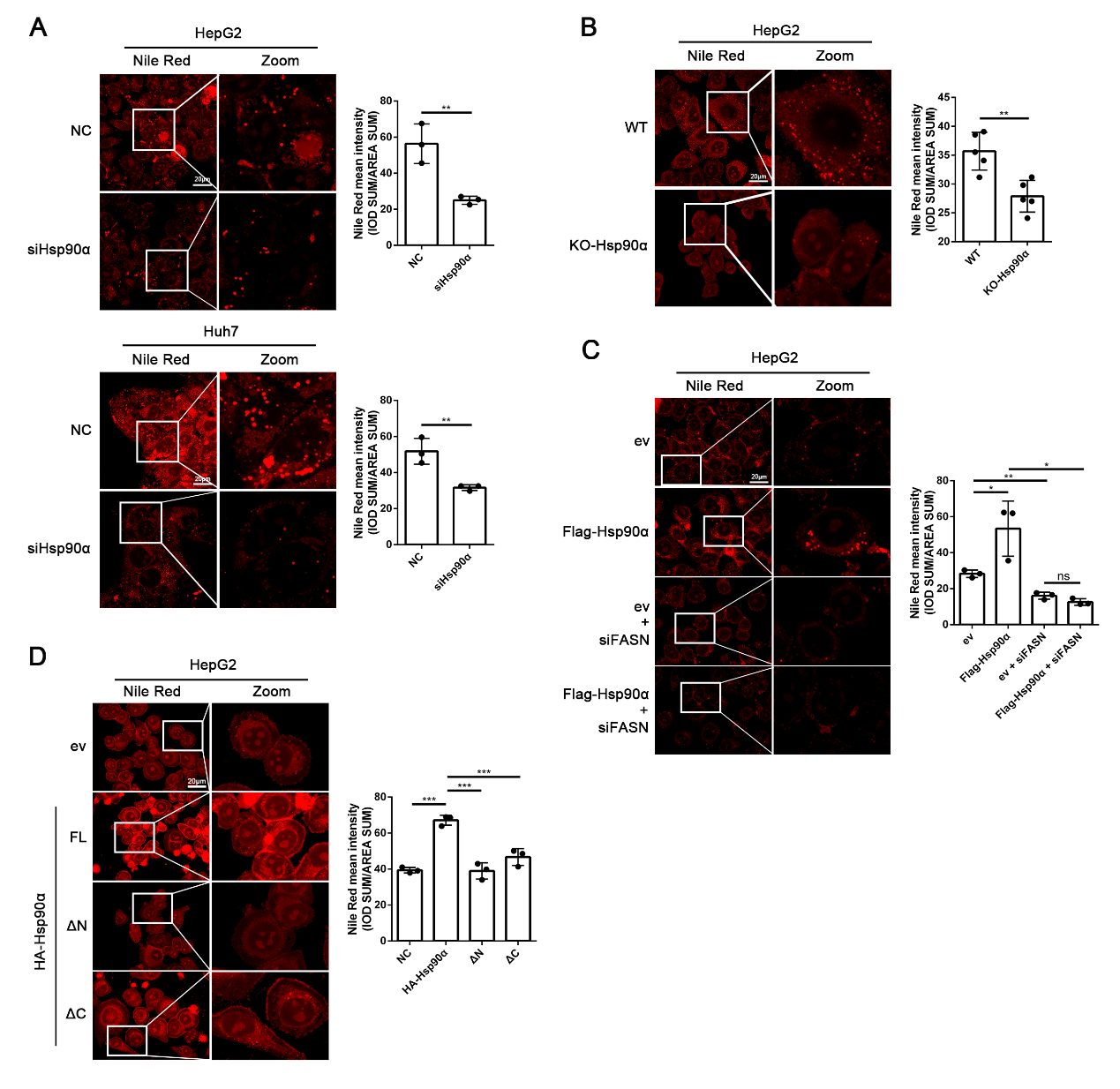
**

**Supplementary Figure 1.** **The** **existence and structural integrity of Hsp90α are required for lipid accumulation in HCC cells.**

Fluorescence analysis of lipid accumulation in HCC cells indicated by Nile Red staining after different treatment as follows: (A) knockdown of Hsp90α; (B) knockout of Hsp90α; (C) overexpression of Hsp90α; (D) transfection with indicated Hsp90a plasmids. Scale bar: 20 μm. Data are shown as means ± SD, n=3 per group, **p* < 0.05, ***p* < 0.01, ****p* < 0.001.

**
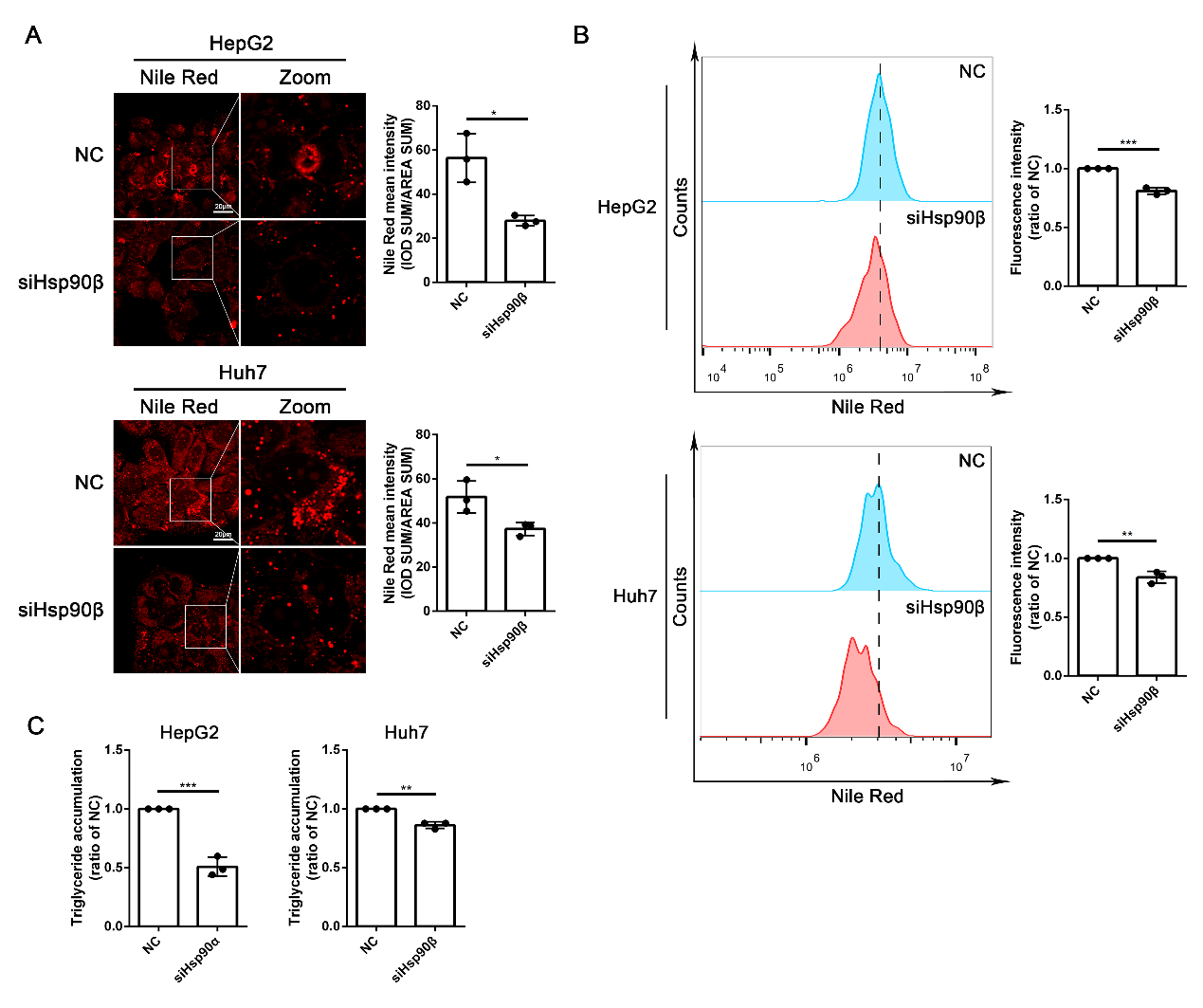
**

**Supplementary Figure 2.** **The lipid accumulation in HCC cells was reduced by Hsp90β knockdown.**

(A) Fluorescence images (left) and quantification (right) of Nile Red staining in HCC cell lines HepG2, Huh7 were shown. Scale bar: 20 μm. Data are shown as means ± SD, n=3 per group, **p* < 0.05. (B) The lipid accumulation levels were detected by flow cytometry with Nile Red. Data are shown as means ± SD, n = 3 per group, ***p* < 0.01, ****p* < 0.001. (C) The cellular TG levels were measured by assay kits. Data are shown as means ± SD, n = 3 per group, ***p* < 0.01, ****p* < 0.001.

**
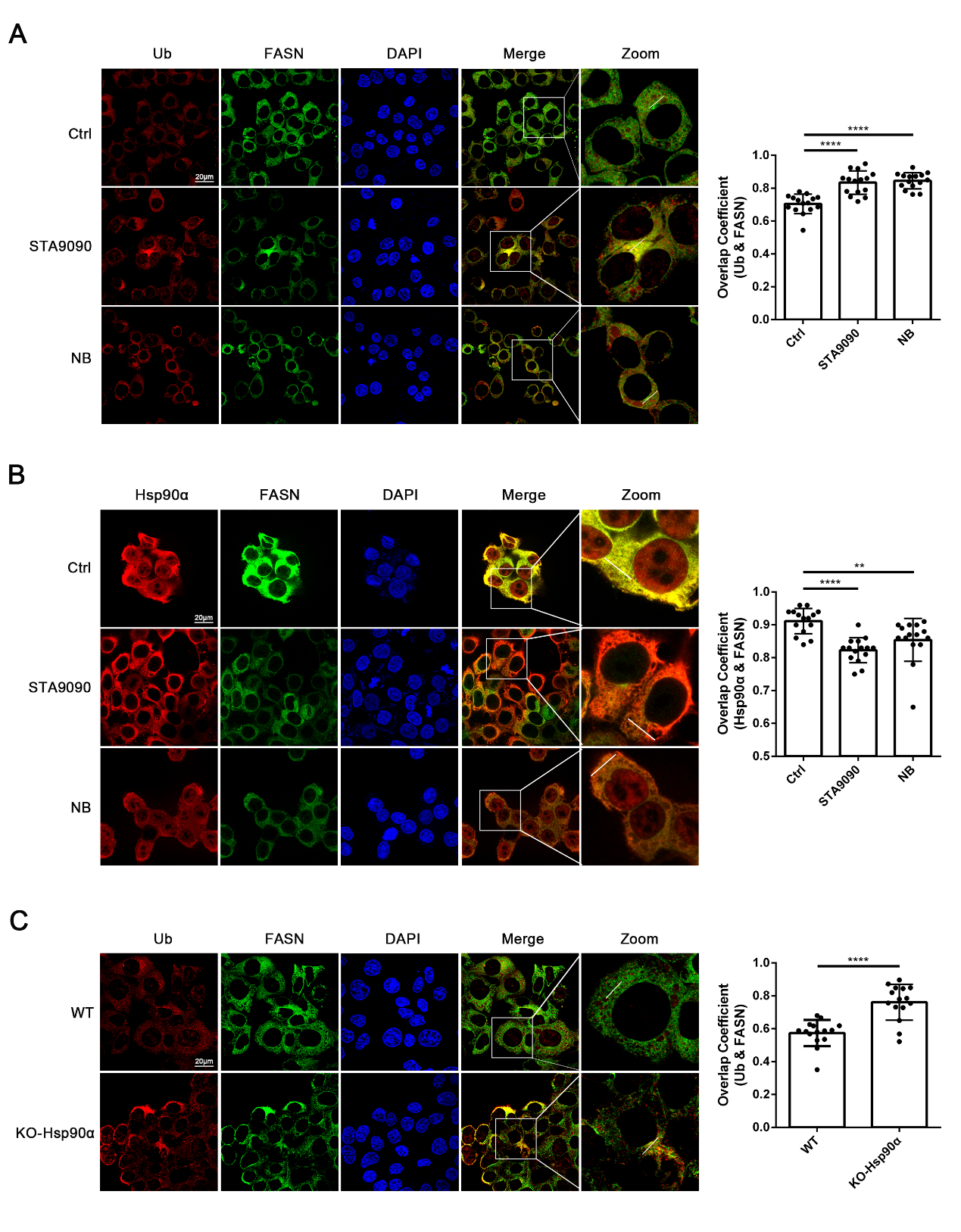
**

**Supplementary Figure 3.** **Inhibition or knockdown of Hsp90α resulted in enhanced co-localization between ubiquitin and FASN, and weakened co-localization between Hsp90α and FASN.**

(A) Immunofluorescence staining for Ub (red), FASN (green), and DAPI (blue) was performed in HepG2 cells treated with DMSO (1 μL DMSO in 1 mL DMEM for 24 h) or STA9090 (100 nM for 24 h) or NB (500 μM for 24 h). Scale bar: 20 μm. Data are shown as means ± SD, n = 15 cells per group, *****p* < 0.0001. (B) Immunofluorescence staining for Hsp90α (red), FASN (green), and DAPI (blue) was performed in HepG2 cells treated with DMSO (1 μL DMSO in 1 mL DMEM for 24 h) or STA9090 (100 nM for 24 h) or NB (500 μM for 24 h). Scale bar: 20 μm. Data are shown as means ± SD, n = 15 cells per group, ***p* < 0.01, *****p* < 0.0001. (C) Immunofluorescence staining for Ub (red), FASN (green), and DAPI (blue) was performed in wild type HepG2 cells and Hsp90α-knockout HepG2 cells. Scale bar: 20 μm. Data are shown as means ± SD, n = 15 cells per group, *****p* < 0.0001.

**
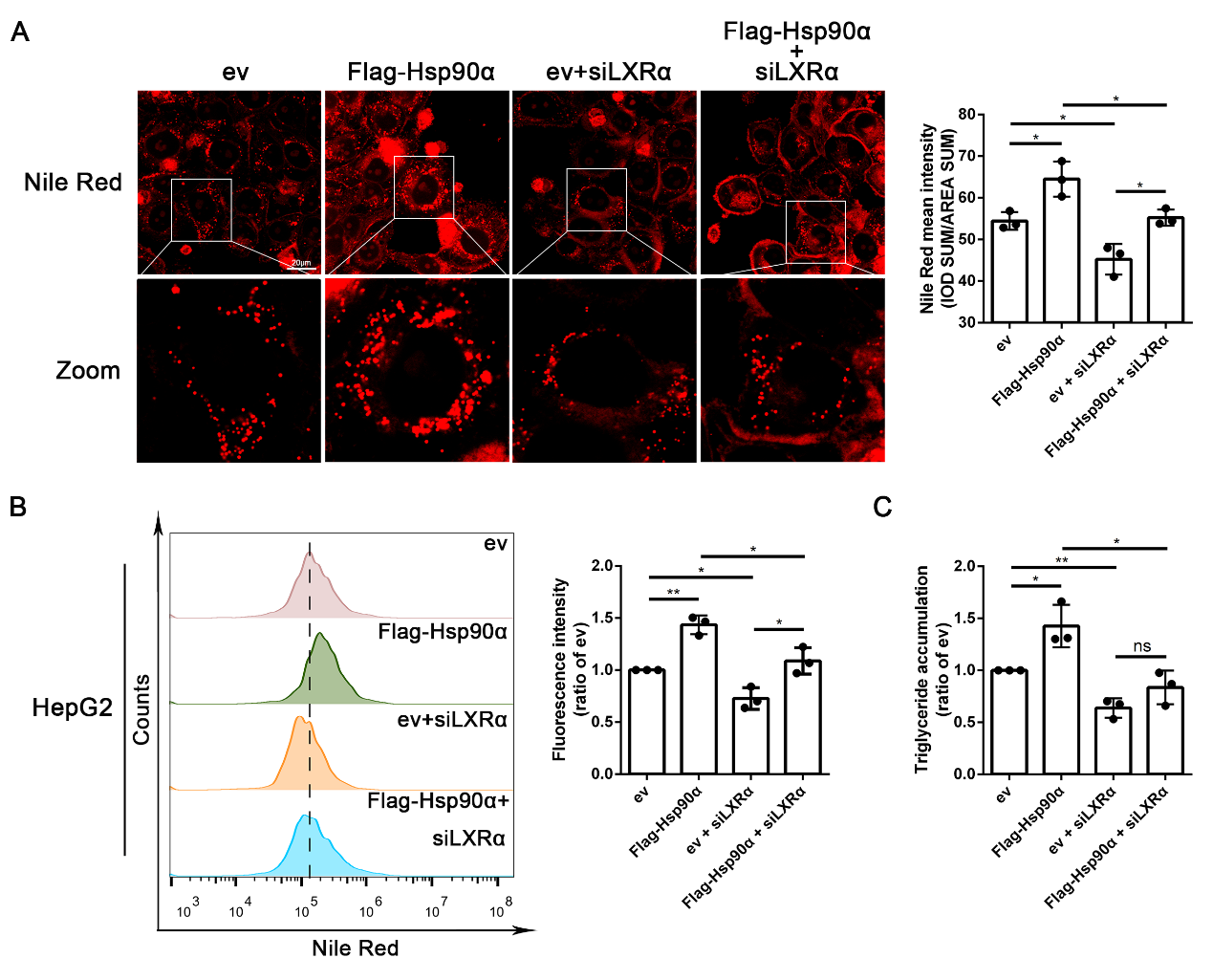
**

**Supplementary Figure 4.** **LXRα is required for Hsp90α-promoted lipid accumulation in HCC cells.**

Lipid accumulation of HepG2 cells after overexpression of Hsp90α combined with or without siLXRα were detected by confocal microscope with Nile Red staining (A), flow cytometry with Nile Red staining (B) and TG assay kits (C). Scale bar: 20 μm. Data are shown as means ± SD, n = 3 per group, **p* < 0.05, ***p* < 0.01.

**
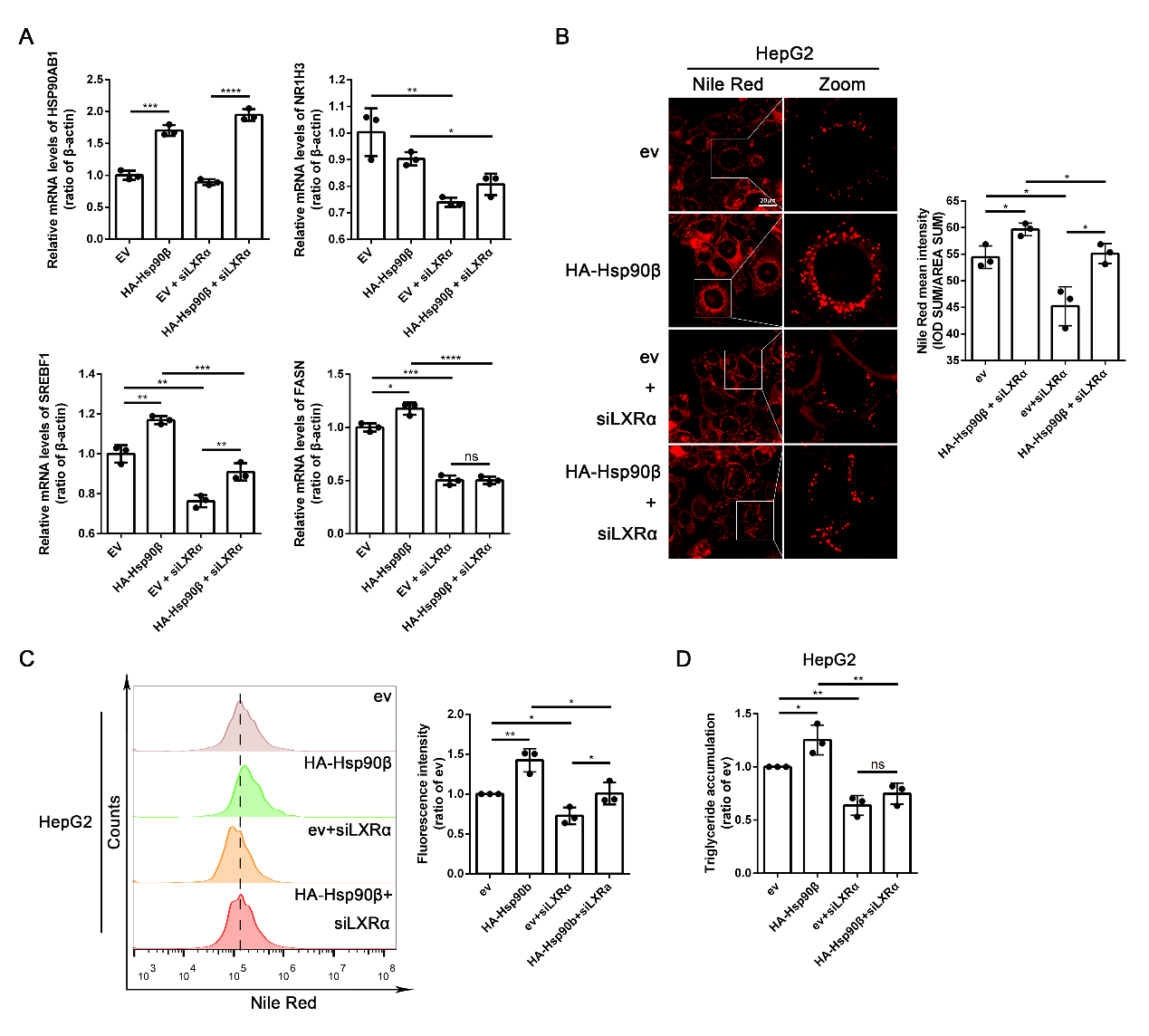
**

**Supplementary Figure 5.** **Hsp90β regulates lipogenesis through the LXRα/SREBP1/FASN axis.**

(A) mRNA levels of HSP90AB1, NR1H3, SREBP1, FASN in HepG2 cells after overexpression of Hsp90β combined with or without siLXRα were detected by qPCR. Data are shown as means ± SD, n = 3 per group, **p* < 0.05, ***p* < 0.01, ****p* < 0.001, *****p* < 0.0001. Lipid accumulation of HepG2 cells after overexpression of Hsp90β combining with or without siLXRα were detected by confocal microscope with Nile Red staining (A), flow cytometry with Nile Red staining (B) and TG assay kits (C). Scale bar: 20 μm. Data are shown as means ± SD, n = 3 per group, **p* < 0.05, ***p* < 0.01.

**Supplemental Tables**

**Supplementary Table 1.** siRNA, sgRNA sequences used in the study.

| siRNA/sgRNA | Sequence (5'-3') |
| --- | --- |
| siHsp90α sense | CCCAGUUGAUGUCAUUGAUTT |
| siHsp90α antisense | AUCAAUGACAUCAACUGGGTT |
| siFASN sense | UGGAGCGUAUCUGUGAGAAdTdT |
| siFASN antisense  Hsp90α sgRNA | dTdTACCUCGCAUAGACACUCUU  ATTAACCCAGAATCGGATTT |

**Supplementary Table 2.** Antibodies used in western blotting.

| Antibodies | Sourse | | Identifier | Dilution |
| --- | --- | --- | --- | --- |
| anti-Hsp90α (Rat) | Enzo | 6251422 | | 1:10000 |
| anti-Hsp90α (Rabbit) | Cell Signaling Technology | 8165s | | 1:1000 |
| anti-LXRα (Rabbit) | Proteintech | 14351-1-AP | | 1:1000 |
| anti-SREBP1 (Mouse)  anti-FASN (Rabbit)  anti-HA (Rabbit)  anti-FLAG (Mouse)  anti-Ubiquitin (Mouse)  anti-β-Tubulin (Rabbit)  anti- Histone-H3 (Rabbit)  anti-β-actin (Mouse)  680RD anti-Rat (Donkey)  680RD anti-Mouse (Donkey)  680RD anti-Rabbit (Donkey)  800RD anti-Mouse (Donkey)  800RD anti-Rabbit (Donkey) | Santa Cruz Proteintech  Sigma-Aldrich  Sungene Biotech Co  Santa Cruz  Cell Signaling Technology  Proteintech  Ray Antibody  IRDye  IRDye  IRDye  IRDye  IRDye | SC-13551  10624-2-AP  H6908  C:KM8002  SC-8017  2128S  17168-1-AP  RM2001  C40304-01  C80522-25  C80605-15  C70301-02  C70405-07 | | 1:500  1:1000  1:1000  1:1000  1:500  1:1000  1:1000  1:1000  1:5000  1:20000  1:20000  1:20000  1:20000  1:20000 |

**Supplementary Table 3**. Primer sequences used in the study.

| Primer name | Sequence (5'-3') |
| --- | --- |
| HSP90AA1 sense | AGGAGGTTGAGACGTTCGC |
| HSP90AA1 antisense | AGAGTTCGATCTTGTTTGTTCGG |
| SREBF1 sense | GCGCCTTGACAGGTGAAGTC |
| SREBF1 antisense | GCCAGGGAAGTCACTGTCTTG |
| FASN sense | GAGGTGTCAGAGAACGGCAA |
| FASN antisense | GTGTCCATGAAGCTCACCCA |
| β-actin sense | CATGTACGTTGCTATCCAGGC |
| β-actin antisense | CTCCTTAATGTCACGCACGAT |

**Supplementary Table 4**. ChIP PCR primer sequences used in the study.

| Primer name | Sequence (5'-3') |
| --- | --- |
| SREBP-1 LXRE region sense | GTAAACGGAGGGTTGGAGC |
| SREBP-1 LXRE region antisense | CTGAATGGGGTTGGGGTTA |
| FASN LXRE region sense | GGCAGCAGCAACCAATC |
| FASN LXRE region antisense | AGCGGCTCCCTTTGTC |
| FASN SRE sense | AGCGGGAGGCTGAAGC |
| FASN SRE antisense | CGCACGAGCATCACCC |

**Supplementary Table 5.** Antibodies used in immunofluorescence assays.

| Antibodies | Sourse | Identifier | Dilution |
| --- | --- | --- | --- |
| anti-Hsp90α (Rat) | Enzo | 6251422 | 1:1000 |
| anti-SREBP1 (Mouse)  anti-FASN (Rabbit)  anti-HA (Rabbit)  anti-Ubiquitin (Mouse)  Alexa Fluor 594 anti-Rat  Alexa Fluor 488 anti-Mouse  Alexa Fluor 488 anti-Rabbit  Alexa Fluor 555 anti-mouse | Santa Cruz Proteintech  Sigma-Aldrich  Santa Cruz  Life Technologies  Life Technologies  Life Technologies  Life Technologies | SC-13551  10624-2-AP  H6908  SC-8017  A-21209  A11001  A31628  A31570 | 1:50  1:100  1:50  1:50  1:200  1:200  1:200  1:200 |

**References**

[61] Tan, W., Zhang, J., Liu, L., Liang, M., Li, J., Deng, Z., Zheng, Z., Deng, Y., Liu, C., Li, Y., Xie, G., Zhang, J., Zou, F., & Chen, X. (2022). Hsp90 Inhibitor STA9090 induced VPS35 related extracellular vesicle release and metastasis in hepatocellular carcinoma. *Translational oncology*, *26*, 101502.
